# Cost‐effectiveness of acceptance and commitment therapy for people living with motor neuron disease, and their health‐related quality of life

**DOI:** 10.1111/ene.16317

**Published:** 2024-04-25

**Authors:** Anju D. Keetharuth, Rebecca L. Gould, Christopher J. McDermott, Benjamin J. Thompson, Charlotte Rawlinson, Mike Bradburn, Matt Bursnall, Pavithra Kumar, Emily J. Turton, Paul Tappenden, David White, Robert J. Howard, Marc A. Serfaty, Lance M. McCracken, Christopher D. Graham, Ammar Al‐Chalabi, Laura H. Goldstein, Vanessa Lawrence, Cindy Cooper, Tracey Young, the COMMEND Collaboration Group

**Affiliations:** ^1^ School of Medicine and Population Health, Sheffield Centre for Health and Related Research University of Sheffield Sheffield UK; ^2^ Division of Psychiatry University College London London UK; ^3^ Sheffield Institute for Translational Neuroscience University of Sheffield Sheffield UK; ^4^ Clinical Trials Research Unit, Sheffield Centre for Health and Related Research University of Sheffield Sheffield UK; ^5^ Priory Hospital North London London UK; ^6^ Department of Psychology Uppsala University Uppsala Sweden; ^7^ Department of Psychological Sciences & Health University of Strathclyde Glasgow UK; ^8^ Maurice Wohl Clinical Neuroscience Institute King's College London London UK; ^9^ Department of Psychology, Institute of Psychiatry, Psychology & Neuroscience King's College London London UK; ^10^ Health Service & Population Research Department, Institute of Psychiatry, Psychology & Neuroscience King's College London London UK

**Keywords:** acceptance and commitment therapy, cost‐effectiveness, health‐related quality of life, motor neuron disease, RCT

## Abstract

**Background:**

Given the degenerative nature of the condition, people living with motor neuron disease (MND) experience high levels of psychological distress. The purpose of this research was to investigate the cost‐effectiveness of acceptance and commitment therapy (ACT), adapted for the specific needs of this population, for improving quality of life.

**Methods:**

A trial‐based cost–utility analysis over a 9‐month period was conducted comparing ACT plus usual care (*n* = 97) versus usual care alone (*n* = 94) from the perspective of the National Health Service. In the primary analysis, quality‐adjusted life years (QALYs) were computed using health utilities generated from the EQ‐5D‐5L questionnaire. Sensitivity analyses and subgroup analyses were also carried out.

**Results:**

Difference in costs was statistically significant between the two arms, driven mainly by the intervention costs. Effects measured by EQ‐5D‐5L were not statistically significantly different between the two arms. The incremental cost‐effectiveness was above the £20,000 to £30,000 per QALY gained threshold used in the UK. However, the difference in effects was statistically significant when measured by the McGill Quality of Life‐Revised (MQOL‐R) questionnaire. The intervention was cost‐effective in a subgroup experiencing medium deterioration in motor neuron symptoms.

**Conclusions:**

Despite the intervention being cost‐ineffective in the primary analysis, the significant difference in the effects measured by MQOL‐R, the low costs of the intervention, the results in the subgroup analysis, and the fact that ACT was shown to improve the quality of life for people living with MND, suggest that ACT could be incorporated into MND clinical services.

## BACKGROUND

Motor neuron disease (MND) is a neurodegenerative disease with median survival estimated at 2–3 years from diagnosis [1]. The rapid progression of the disease poses considerable psychological burden on people living with MND (plwMND) leading to significant changes to daily living and a shortened lifespan which can also result in psychological distress [2, 3]. The prevalence of depression for plwMND varies between 10% and 45% and a recent meta‐analysis from 46 studies estimates the pooled prevalence to be 34% [4]. Anxiety is prevalent not only at the time of diagnosis but also as the disease progresses [3]. A psychological intervention, acceptance and commitment therapy (ACT), adapted for the specific needs of plwMND [5] was delivered as part of a multicentre randomised controlled trial (RCT) conducted in the UK where healthcare provided by the National Health Service (NHS) is free at the point of use. The primary aim of this study was to assess the cost‐effectiveness of ACT plus usual care (UC) compared to UC alone for plwMND. While the intervention has been shown to be clinically effective [6], it is important to assess whether it represents value for money for the NHS. The secondary aim was to analyse the health‐related quality of life of plwMND as the disease progresses.

## METHODS

### Study design and participants

Full details of the study design and trial results are described elsewhere [6, 7]. In summary, our cost‐effectiveness analysis was based on 191 plwMND who participated in a multicentre, parallel RCT comparing ACT plus UC (*n* = 97) with UC alone (*n* = 94). PlwMND were recruited from 16 centres/clinics across England, Wales and Scotland from September 2019 to August 2022. Measurements for the trial were recorded prior to randomisation at baseline and at 6 and 9 months post‐randomisation. Despite COVID‐related restrictions, the follow‐up rates were 82% (*n* = 156) and 71% (*n* = 136) at 6 and 9 months, respectively. Thirteen plwMND died between randomisation and 6 months and another 10 died between the 6‐ and 9‐month follow‐ups. At the time of randomisation, plwMND were aged between 28 and 92 years, with a mean (standard deviation [SD]) age of 63 (11) years; 111 subjects were male and 80 were female.

### The intervention

PlwMND attended up to eight 1:1 sessions of ACT, each lasting up to 1 h, over the course of 4 months, with a minimum of four sessions being delivered face‐to‐face within a clinic environment, participant's home or via videoconference/telephone depending on patient preference and therapist availability with sessions supplemented by online audio/compact discs. Each participant saw the same therapist for the duration of the intervention. Therapists comprised 31 clinical psychologists, accredited cognitive behavioural therapists, counselling psychologists, counsellors and psychotherapists. Therapists received fortnightly group supervision via telephone or videoconference from an ACT‐trained clinical psychologist or psychotherapist. In addition, therapists attended 4 days of training prior to delivery and a 1‐day top‐up approximately 12 months later. UC mainly comprised medication for managing MND and MND‐related symptoms as outlined in the National Institute for Health and Care Excellence (NICE) MND Clinical Guideline [8] but could also included counselling or other therapy.

This trial was pre‐registered with the International Standard Randomised Controlled Trial Registry (ISRCTN12655391) and was approved by the London‐Dulwich Research Ethics Committee, Health Research Authority and Health and Care Research Wales (19/LO/0272). All participants provided fully informed consent.

A health economics analysis plan (HEAP) [9] (Appendix S1: SM1) was written and approved by the Trial Steering Committee before the analysis stage. Stata version 18 [10] was used for the cost‐effectiveness analysis. Analyses are reported using the Consolidated Health Economic Evaluation Reporting Standards (CHEERS) checklist [11] (Appendix S1: SM2).

### Outcomes

We used the self‐administered EQ‐5D‐5L [12] to assess health‐related quality of life (mobility, self‐care, usual activities, pain and discomfort, and anxiety and depression) at baseline, 6 months and 9 months. At the domain level, items are scored from levels 1 to 5 where 1 and 5 are the least and most impaired quality of life, respectively. In the absence of a value set for the UK and in line with NICE recommendations, we used mapped tariffs from EQ‐5D‐5L to EQ‐5D‐3L [13]. The primary outcome used in the trial was the McGill Quality of Life‐Revised (MQOL‐R) [14] questionnaire at 6 months post‐randomisation. The MQOL‐R consists of 15 items on a Likert scale (0–10) and we used the total score from 14 questions comprising the four domains (existential – four items, psychological – four items, physical – three items, social – three items). Subscale scores are the means of the constituent items and the total score is the mean of the subscale scores where a high score indicates the ‘best’ case.

### Type of evaluation, perspective and length of study

The primary health economic analysis was a cost–utility analysis comparing differences in costs with differences in EQ‐5D‐5L scores across the intervention and control arms for the study period of 9 months and adopted an NHS and Personal Social Services (PSS) perspective as per the NICE Reference Case [15]. We used the area under the curve to generate quality‐adjusted life years (QALYs) over a 9‐month period and a utility score of 0 was assigned on the date of death for the purpose of QALY computation. A secondary analysis was undertaken with differences in effectiveness measured by MQOL‐R scores over the 9‐month period (see deviation to HEAP in Appendix S1: SM1). An existing cohort‐level state transition Markov model [16] informed by data on the King's clinical staging [17, 18] and progression, mortality, EQ‐5D‐3L and resource use from the ALS‐CarE study was used to explore the cost‐effectiveness of ACT plus UC over the life course of the disease. Separate analyses were conducted to explore alternative assumptions regarding the duration of the benefit of the intervention of health‐related quality of life (additional details are provided in Appendix S1: SM3).

### Resource use and unit costs

Resource use data for each participant covered a retrospective period of 6 months at baseline. At 6 and 9 months, data were collected for a retrospective period of 6 and 3 months, respectively, using a modified version of the Client Service Receipt Inventory (CSRI) [19]. Participants were asked about their use of primary and community services; hospital, nursing homes or hospice inpatient services; outpatient and day care services; equipment; home adaptation services; and psychological services. For the latter three categories, participants also reported whether these were provided or funded by the NHS, local authorities, charities or the participant themselves.

All unit costs were estimated at 2021/2022 prices and were collected from various sources as shown in Table 1 (Appendix S1: Tables A1a–f, SM4). Unit costs from earlier years were inflated to 2021/2022 using the NHS Cost Inflation Index (NHSCII) [20].

**TABLE 1:** Sources of resource use costs.

Source of costs	Services used
I. Primary and Community services	
PSSRU 2022	General practitioner, social worker, psychologist, psychotherapy, counselling, home help (personal care) MNDA advisor, MNDA volunteer visitor, home help (household tasks), sitting service (charity provider)
National schedule of NHS costs 2021/2022	Physiotherapist, occupational therapist, speech and language therapist, dietician, nutrition nurse, district nurse, palliative care nurse, nurse specialist (MND, respiratory)
II. Hospital, nursing home, hospice and inpatient services	
PSSRU 2022	Nursing or residential home, hospice including respite care, inpatient ward, intensive care unit, admission for gastronomy tube insertion/management, admission for NIV/IV assessment/management, other elective inpatients
National schedule of NHS costs 2021/2022	
III. Outpatient and day care services	
National schedule of NHS costs 2021/2022	Neurology patient ward, other patient wards, A&E visit
IV. Equipment	
Commercial webpages	Ankle/foot orthotic, walking aids, wheelchair (manual and electric), mobile arm support, Lightwriter, speech amplifier, stairlift, specialist cutlery, rise recliner chair, specialist bed, mattress elevator, hoist (bedroom), wash and dry toilet, bath hoist, neck support, specialist computer equipment, environmental controls, feeding pump, NIV
V. Home adaptations	
PSSRU 2020	Extension built, downstairs toilet installed, downstairs shower installed, wheelchair ramps installed, doors widened, bathroom adapted, through floor lift/elevator, handrails installed
VI. Psychological therapies	
PSSRU 2022	Cognitive behavioural therapy, relaxation therapy, mindfulness‐based therapies, counselling, psychodynamic therapy

Abbreviations: A&E, accident and emergency; IV, invasive ventilation; MND, motor neuron disease; MNDA, Motor Neurone Disease Association; NHS, National Health Service; NIV, non‐invasive ventilation; PSSRU, Personal Social Services Research Unit.

### Statistical analysis

The primary analysis was carried out on an intention‐to‐treat (ITT) basis comparing costs and QALYs over 9 months. Missing data were imputed by treatment arm using chained equations to create 100 complete datasets [21]. Regression analysis on total costs and total QALYs was used to adjust for clinically relevant baseline covariates [22]. A bivariate multilevel analysis was carried out using seemingly unrelated regression equations for the cost and effectiveness components of the analysis. Estimating costs and effects jointly ensures that their correlation is incorporated appropriately [23]. An incremental analysis was undertaken by dividing the mean incremental costs by the mean incremental QALYs to produce an incremental cost‐effectiveness ratio (ICER). To assess the uncertainty associated with the estimates, cost‐effectiveness acceptability curves (CEACs) were used to illustrate the probability of each treatment being most cost‐effective for a range of threshold values.

### Sensitivity and subgroup analysis

Deterministic sensitivity analyses were carried out to test the impact of data, assumptions and analysis methods on results. First, we conducted a per protocol analysis which included participants in the intervention arm who had received at least four sessions within a 4‐month period and prior to the 6‐month follow‐up visit. Second, a complete case analysis was undertaken including only participants who had no missing data for costs and outcomes. Third, a partial societal perspective was taken with respect to costs to include costs incurred by the voluntary sector and by plwMND. To investigate the impact of heterogeneity on the cost‐effectiveness of the intervention, the following subgroups were considered: severity of condition at baseline using Amyotrophic Lateral Sclerosis Functional Rating Scale‐Revised questionnaire (ALSFRS‐R) [24]; severity of baseline depression and anxiety using the Modified Hospital Anxiety and Depression Scale depression component (M‐HADS‐D) and anxiety component (M‐HADS‐A) [25, 26]; average rate of deterioration per month measured by the ALSFRS‐R pre‐slope to measure decline in MND functionality between symptom onset and baseline; and participants recruited during COVID‐19 pandemic‐related lockdown restrictions versus those recruited pre‐and post‐restrictions.

## RESULTS

### Missing data

Missing data for total QALYs for plwMND at 9 months was 19% and 16% in the intervention and control arms, respectively, and missing data for total costs at 9 months was 30% for each arm. Age, gender, ALSFRS‐R scores and MQOL‐R scores were found to be predictors of missing QALYs and costs ruling out the assumption that data were missing completely at random. Multiple imputation of total costs and QALYs was based on the predictors of missingness.

### Health‐related quality of life of plwMND

The EQ‐5D‐5L scores decreased as the stage of the disease progressed over time (Table 2). The mean (SD) score EQ‐5D‐5L scores fell from 0.667 (0.184) in Stage 1 to 0.282 (0.362) in Stage 4. At the domain level, the scores for the self‐care domain worsened most from Stage 1 (mean 1.8, SD 0.93) to Stage 4 (mean 3.52, 1.33). The least impaired domain was anxiety and depression in Stage 1. The mean (SD) scores for anxiety and depression were 1.74 (0.77) in Stage 1, 1.62 (0.85) in Stage 2, 1.67 (0.85) in Stage 3 and 2.11 (0.94) in Stage 4. At baseline, 75% and 81% of participants reported either Level 1 or 2 combined for the anxiety and depression dimension in the intervention and control arms, respectively (Appendix S1: Table A2, SM4).

**TABLE 2:** EQ‐5D‐5L utility scores, domain scores and McGill Quality of Life‐Revised (MQOL‐R) scores by King's clinical stage.

Outcome measures	Stage 1 (*n* = 75)		Stage 2 (*n* = 129)		Stage 3 (*n* = 205)		Stage 4 (*n* = 73)	
	Mean	SD	Mean	SD	Mean	SD	Mean	SD
EQ‐5D‐5L scores	0.667	0.184	0.561	0.249	0.404	0.284	0.282	0.362
Mean levels by EQ‐5D‐5L domains								
Mobility	2.56	1.32	2.72	1.33	3.44	1.16	3.48	1.5
Self‐care	1.8	0.93	2.4	1.2	3.1	1.25	3.52	1.33
Usual activity	2.25	0.95	2.74	1.1	3.31	1.08	3.67	1.25
Pain/discomfort	1.54	0.64	1.88	0.89	2.2	0.9	2.36	1.02
Anxiety/depression	1.74	0.77	1.62	1.62	1.67	0.85	2.11	0.94
MQOL‐R scores	7.25	1.33	6.76	1.61	6.59	1.57	5.97	1.40

*Note*: Data from all individuals at the various timepoints have been pooled. Abbreviations: MQOL‐R, McGill Quality of Life‐Revised; SD, standard deviation.

### Resource use and costs

Resource use as described in Table 3 shows no significant differences in the mean resource use between the intervention and control arms (more details in Appendix S1: Tables A3–A4, SM4). The majority of the resource use costs were accounted for by primary and community services with mean costs per person in the intervention arm of £1754 (95% CI £1373 to £2135) and £1666 (95% CI £970 to £2362) in the control arm. Although only 10 and 14 plwMND in the intervention and control arms, respectively, used inpatient services (hospital, nursing home, hospice), this accounted for the second highest costs (Appendix S1: Tables A5–A6, SM4). Home adaptation costs were borne by charities and individuals funding themselves and the mean (SD) was £592 (£1910). Intervention costs were relatively small (mean £712; 95% CI £669 to £756) with intervention delivery and ongoing therapist supervision accounting for 70% and 25% of the total intervention costs, respectively, with the remaining 5% for initial training of therapists (more details in Appendix S1: Table A7, SM4).

**TABLE 3:** Mean resource use over the 9‐month period by treatment arm.

Resource use categories	Intervention	Control	*P* value
	Mean (95% CI)	Mean (95% CI)	
Primary and community services	31.38^a^ (20.68 to 42.09]	29.14 (11.00 to 47.27)	0.19
Hospital, nursing home or hospice services (visits)	0.27 (0.13 to 0.41]	0.26 (0.12 to 0.39)	0.99
Hospital, nursing home or hospice services (total nights)	1.50 (0.36 to 2.65]	0.92 (0.32 to 1.51)	0.85
Outpatient and day care services (number of attendances)	1.64 (1.17 to 2.16]	2.48 (1.45 to 4.23)	0.26
Equipment (NHS, LA, charities and self‐funded)	3.84 (3.03 to 4.66]	3.49 (2.81 to 4.16)	0.91
Home adaptations	1.30 (0.82 to 1.77]	0.86 (0.58 to 1.14)	0.69
Psychological therapies	2.40 (0.76 to 4.04]	0.79 (0.28 to 1.30)	0.22

Abbreviations: CI, confidence interval; LA, local authorities; NHS, National Health Service.

^a^ The interpretation of this figure is that on average people in the intervention group used primary and community services 31 times over 9 months.

### Cost‐effectiveness analysis

#### Primary analysis

**FIGURE 1:**
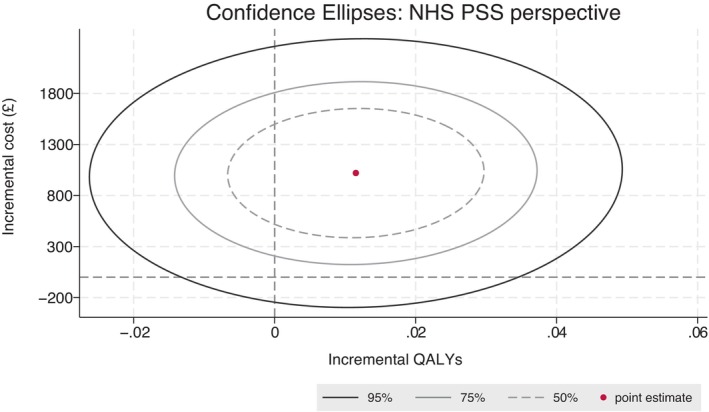
Cost‐effectiveness plane: primary analysis. NHS, National Health Service; PSS, Personal Social Services; QALY, quality‐adjusted life year.

**FIGURE 2:**
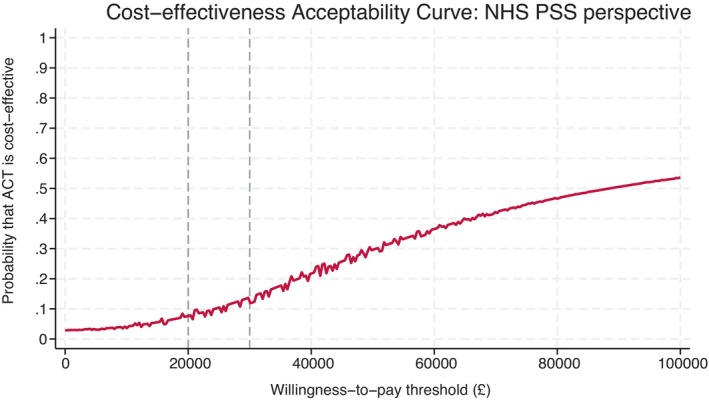
Cost‐effectiveness acceptability curve: primary analysis. ACT, acceptance and commitment therapy; NHS, National Health Service; PSS, Personal Social Services.

The mean difference in EQ‐5D‐5L scores was not statistically significant at all three timepoints (Table 4). A decline in EQ‐5D‐5L scores was observed in both the intervention and control arms at both the 6‐ and 9‐month follow‐ups. The mean difference in imputed QALYs over 9 months between the intervention and control arms was 0.019 (95% CI −0.07 to 0.05), which was not statistically significant. From the regression model, the mean total costs were higher in the intervention arm, but the mean difference was not statistically significant at the 5% level (mean difference = £1019; 95% CI −£34 to £2074). The point estimate of the ICER is £88,507, which is above the £20,000 to £30,000 per QALY gained threshold commonly used by NICE in the UK for decision‐making [15]. Figure 1 shows that the 95% confidence ellipse extends into the top two quadrants reflecting the uncertainty about the QALY gains and the fact that the costs of the intervention are consistently greater than those for the comparator. The associated CEAC in Figure 2 estimates that the probability that ACT is cost‐effective is only 8% at the willingness‐to‐pay threshold of £20,000.

**TABLE 4:** Mean costs and outcomes after imputing missing values used in the primary analysis.

Costs per participant	Intervention	Control	Mean difference (95% CI)	*P* value
	Mean (SE) (*n* = 97)	Mean (SE) (*n* = 94)		
Intervention costs	712 (22)		712 (668 to 756)	<0.001
Resource use costs	3843 (406)	3413 (489)	−185 (−1361 to 990)	0.755
Total mean costs	4555 (403)	3413 (401)	1142 (16 to 2269)	0.047
Mean utilities and QALYs				
EQ‐5D‐5L scores at baseline	0.509 (0.029)	0.533 (0.028)	−0.024 (−0.103 to 0.056)	0.556
EQ‐5D‐5L scores at 6 months	0.418 (0.036)	0.427 (0.034)	−0.008 (−0.106 to 0.089)	0.864
EQ‐5D‐5L scores at 9 months	0.379 (0.035)	0.378 (0.036)	0.000 (−0.098 to 0.099)	0.996
QALY for 9 months	0.33 (0.02)	0.34 (0.02)	−0.01 (−0.07 to 0.05)	0.719

*Note*: These figures are the observed means after imputing missing data that have not yet been adjusted for baseline covariates (see Table 5 for baseline‐adjusted estimates).

Abbreviations: QALY, quality‐adjusted life year; SE, standard error.

#### Secondary analysis

**FIGURE 3:**
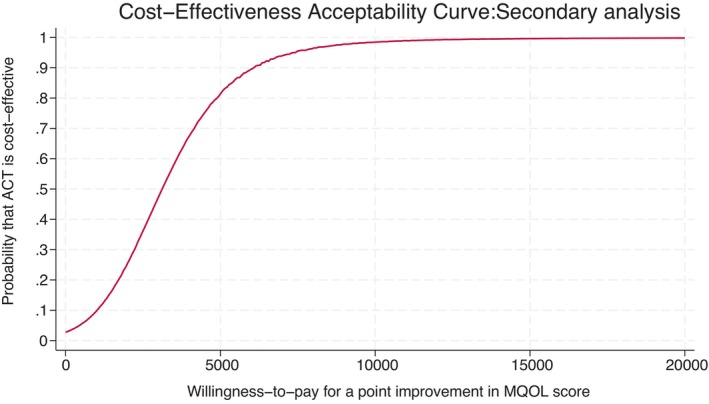
Cost‐effectiveness acceptability curve: secondary analysis. ACT, acceptance and commitment therapy; MQOL, McGill Quality of Life.

In the secondary analysis, the regression resulted in a statistically significant mean difference in MQOL‐R scores of 0.34 (95% CI 0.15 to 0.52) over 9 months. To our knowledge, there are no monetary values to incremental changes in MQOL‐R scores to provide any guidance on the appropriate willingness‐to‐pay values. The CEAC in Figure 3 shows the probability estimates for a range of implicit monetary values associated with a unit improvement in the MQOL‐R score. The intervention has an 85% probability of being cost‐effective if the NHS is willing to pay £5000 for a unit improvement in the MQOL‐R score. This probability rises to 100% if the NHS is willing to pay £10,000 for a unit improvement in the MQOL‐R score.

Analyses from the long‐term modelling indicate that the cost‐effectiveness of the intervention is heavily dependent on the duration over which its benefits are sustained. Assuming a treatment benefit duration of 9 months, the probabilistic ICER was estimated to be £96,654 per QALY gained. If the treatment effect is assumed to persist for 5 years, the probabilistic ICER is estimated to be substantially lower, at £32,314 per QALY gained. Further details on the results of the modelling analysis are provided in Appendix S1: SM3.

#### Sensitivity analysis

The sensitivity analyses show that the results of the primary analysis are robust. In the per protocol analysis where only those individuals who completed at least four sessions were included, the point estimate of the ICER is lower at £47,582 but still above the threshold of being cost‐effective (Table 5). The intervention remains cost‐ineffective if we use complete data without multiple imputation. The societal analysis taking into consideration the costs incurred by the voluntary sector and plwMND yielded an even smaller probability of the intervention being cost‐effective. In all the analyses, the incremental costs are statistically significant and QALYs remain not significant at the 5% level. Given the results of the within‐trial analyses, the results of the long‐term modelling are reported in Appendix S1: SM3. Detailed results on the aforementioned analyses can be found in Appendix S1: Tables A8–A17, Figures A1–A6, SM4.

**TABLE 5:** Summary results from primary, secondary and sensitivity analyses.

Summary results	Primary analysis	Secondary analysis	Sensitivity analysis with EQ‐5D‐5L as outcome		
	ITT using EQ‐5D‐5L as outcome	ITT using MQOL‐R as outcome	Complete case	Per protocol	Partial societal perspective
Difference in mean (95% CI) intervention costs £	712 (668 to 756)	712 (668 to 756)	790 (764 to 816)	802 (782 to 821)	712 (668 to 756)
Difference in mean (95% CI) resource use costs £	1142 (16 to 2269)	1142 (16 to 2269)	592 (−589 to 1772)	568 (−630 to 1766)	3254 (1156 to 5353)
Difference in mean (95% CI) total costs £	1019 (−34 to 2074)	1019 (−34 to 2074)	522 (−596 to 1640)	539 (−595 to 1664)	2927 (1020 to 4834)
Difference in QALY (95% CI) at 9 months	0.012 (−0.019 to 0.042)	0.34^a^ (0.15 to 0.52)	0.008 (−0.022 to 0.036)	0.011 (−0.017 to 0.039)	0.012 (−0.018 to 0.043)
ICER £	£88,507	£3120^b^	£72,233	£47,582	£241,093
Probability that ACT is cost‐effective at £20,000/£30,000	8%	Probability that ACT is cost‐effective if NHS is willing to pay £5000 (£10,000) for a point improvement in MQOL‐R score = 85% (100%)	26%	30%	<1%
Change in outcome needed for ACT to be cost‐effective	0.051		0.022	0.027	0.163

*Note*: Difference is calculated as (ACT + usual care) minus usual care.

Abbreviations: ACT, acceptance and commitment therapy; CI, confidence interval; ICER, incremental cost‐effectiveness ratio; ITT, intention‐to‐treat; MQOL‐R, McGill Quality of Life‐Revised; NHS, National Health Service; QALY, quality‐adjusted life year.

^a^ For the secondary analysis, outcome is measured as MQOL‐R scores.

^b^ There are no monetary values attached to incremental changes in MQOL‐R scores.

#### Subgroup analyses

Subgroup analyses could only be conducted in two of the planned analyses due to the size of subgroups consisting of fewer than 50 plwMND (Appendix S1: Table A18, SM4). The subgroup analyses employed the same methods as for the primary analysis. For plwMND with minimal to mild severity and those with mild to moderate severity (ALSFRS‐R scores), the probability of the intervention being cost‐effective was 58% and 35%, respectively. In the second subgroup analysis based on the rates of deterioration, the difference in costs and QALYs were not statistically significant at the 5% level. However, the results show that the intervention is cost‐effective for the group with medium deterioration (*n* = 64) with a point estimate of an ICER of £13,817. When uncertainty is taken into account, the probability of the intervention being cost‐effective is 86% at a willingness to pay threshold of £20,000/£30,000 per QALY gained. The intervention is not cost‐effective for those with the lowest and highest deterioration rates (more detailed results in Appendix S1: Tables A19–A20, Figures A7–A16, SM4).

## DISCUSSION

This is the first study exploring the cost‐effectiveness of ACT plus UC versus UC alone in plwMND. The incremental benefits measured by EQ‐5D‐5L are very small, which result in a very low probability of ACT plus UC being cost‐effective. Sensitivity analyses show that the results are robust to using multiple imputation, adopting a partial societal perspective, and only including those individuals who completed at least four sessions of the intervention within 4 months. EQ‐5D‐5L was the only pre‐planned measure of effectiveness, but clinical effectiveness was demonstrated in the primary clinical outcome of MQOL‐R. We undertook post hoc analyses using MQOL‐R in which the probability of ACT being cost‐effective was over 85% if the NHS was willing to pay £10,000 for a point improvement. While there is no agreed clinically important difference in MQOL‐R, an 0.5 SDs effect at 6 and 9 months translates to an area under the curve of 0.25 SDs or 0.35 MQOL‐R points. In terms of subgroup analysis, while the intervention was not cost‐effective for those with mild to moderate severity, the probability of being cost‐effective increased at the £20,000/£30,000 threshold per QALY gained. However, the intervention was shown to be cost‐effective for plwMND experiencing a medium rate of deterioration with a probability of 86% of being cost‐effective at the £20,000/£30,000 threshold per QALY gained. This was driven by both a reduction in costs and an increase in effectiveness.

The COMMEND trial raises questions around the suitability of EQ‐5D‐5L to capture the effectiveness of a psychological intervention such as ACT in plwMND in an economic evaluation. EQ‐5D‐5L was used in this trial as it is the measure recommended by NICE's reference case in the UK and a deviation to the reference case is permissible when there is evidence that it is not psychometrically valid in the population of interest. In the absence of such evidence and given that both EQ‐5D‐3L and EQ‐5D‐5L have been validated and used in several cost‐effectiveness analyses involving plwMND [27, 28], there was no justification to use a different measure of effectiveness in the cost‐effectiveness analysis. However, EQ‐5D‐5L fails to capture the benefits of ACT for three main reasons. First, EQ‐5D‐5L has only one item on anxiety and depression whereas 11 of 14 (70%) items used to generate the MQOL‐R score measure psychological health across three “mental health” domains with only three items measuring physical health. Furthermore, some of the items in the MQOL‐R overlap with ACT principles (e.g., those that focus on living a meaningful life and achieving life goals). Therefore, MQOL‐R is better at capturing the effects of the ACT intervention compared with EQ‐5D‐5L. Second, given the nature of MND, as the disease progressed over time, plwMND experienced declining health in the physical health domains of the EQ‐5D‐5L (mobility, self‐care, usual activities, pain and discomfort). Third, at baseline we observed ceiling effects on the anxiety and depression domain with 75% of plwMND reporting either Level 1 or 2 in the intervention arm and 81% in the control arm. This leaves little room for improvement in this domain, which is the main and most direct target of the intervention. However, these ratings are not surprising given that the commissioning brief for this study's funding call explicitly stated that plwMND should not be recruited on the basis of presence of low mood, anxiety or other mental health problem.

This study has shed light on the health‐related quality of life of plwMND. There is a marked decrease in EQ‐5D‐5L scores from Stage 1 to subsequent stages and similar findings are reported in other studies [29]. The EQ‐5D‐5L scores in the COMMEND trial were lower than in the Trajectories of Outcomes in Neurological Conditions (TONiC) study, which recruited patients from 22 MND clinics in a sample with a mean age of 65.07 years [27]. The EQ‐5D‐5L norm for a member of the general population aged between 60 and 65 years is 0.776 and 0.803 for females and males [30], respectively, compared with a within‐trial mean of 0.667 (95% CI 0.625 to 0.709) for Stage 1 and mean of 0.282 (95% CI 0.197 to 0.366) in Stage 4.

The two main components of the costs were resource use and intervention costs. In the cost‐effectiveness analysis, the modelled costs were higher in the intervention group and this difference was statistically significant at the 5% level. This is mainly due to the intervention cost. The difference in the costs of resource use was minimal across the two arms and introduced more uncertainty in the results. As is generally the case, resource use is often skewed by a few participants incurring very high costs but this is a true reflection of what happens in practice. The partial societal perspective analysis showed the costs borne by the voluntary sector and the plwMND. In addition to the physical and psychological burden, MND also imposes financial burden on people.

The main strength of this analysis is the internal validity of the data with costs and benefits being collected as part of an RCT. The analysis was carried out in line with a pre‐specified HEAP with the main deviation being the addition of a secondary analysis with the effects being measured by the primary outcome MQOL‐R. The main weakness of the study is the inability of EQ‐5D‐5L to capture the change in psychological health in plwMND. The trial was not powered on the health economics and therefore this is one reason why we may not see any difference in the outcomes.

The main recommendations are first to systematically review the psychometric properties of EQ‐5D‐5L in psychological interventions, which will help guide the choice of outcome measures used in the cost‐effectiveness of ACT trials. Second, a non‐disease‐specific outcome like MQOL‐R could be used without estimating QALYs. Third, as MQOL‐R is a very widely used questionnaire, a set of preference weights could be developed that would allow the computation of QALYs. While statistical mapping is always a second‐best solution, a mapping algorithm to predict QALYs from MQOL‐R could also be developed.

## AUTHOR CONTRIBUTIONS

**Anju D. Keetharuth:** Formal analysis; methodology; visualization; writing – original draft; writing – review and editing. **Rebecca L. Gould:** Conceptualization; data curation; formal analysis; funding acquisition; investigation; methodology; project administration; supervision; writing – review and editing. **Christopher J. McDermott:** Conceptualization; funding acquisition; investigation; methodology; supervision; writing – review and editing. **Benjamin J. Thompson:** Investigation; methodology; project administration; resources; writing – review and editing. **Charlotte Rawlinson:** Investigation; methodology; project administration; resources; writing – review and editing. **Mike Bradburn:** Conceptualization; formal analysis; funding acquisition; methodology; supervision; writing – review and editing. **Matt Bursnall:** Formal analysis; visualization; writing – review and editing. **Pavithra Kumar:** Investigation; methodology; project administration; resources; writing – review and editing. **Emily J. Turton:** Data curation; software; writing – review and editing. **Paul Tappenden:** Visualization; formal analysis; writing – review and editing; writing – original draft. **David White:** Conceptualization; funding acquisition; methodology; supervision; writing – review and editing. **Robert J. Howard:** Conceptualization; funding acquisition; methodology; supervision; writing – review and editing. **Marc A. Serfaty:** Conceptualization; funding acquisition; methodology; supervision; writing – review and editing. **Lance M. McCracken:** Conceptualization; funding acquisition; methodology; supervision; writing – review and editing. **Christopher D. Graham:** Conceptualization; funding acquisition; methodology; supervision; writing – review and editing. **Ammar Al‐Chalabi:** Conceptualization; funding acquisition; methodology; supervision; writing – review and editing. **Laura H. Goldstein:** Conceptualization; funding acquisition; methodology; supervision; writing – review and editing. **Vanessa Lawrence:** Conceptualization; funding acquisition; methodology; supervision; writing – review and editing. **Cindy Cooper:** Conceptualization; funding acquisition; methodology; supervision; writing – review and editing. **Tracey Young:** Conceptualization; investigation; methodology; formal analysis; supervision; writing – original draft; writing – review and editing.

## FUNDING INFORMATION

This study was funded by the National Institute for Health and Care Research (NIHR) Health Technology Assessment Programme (grant number 16/81/01) and the Motor Neuron Disease Association (grant number Gould/Jul17/936–794). The views expressed are those of the authors and not necessarily those of the National Health Service, the NIHR or the Department of Health and Social Care. The NIHR commissioned the research and had an initial role in stipulating brief details about the study design, but was otherwise not involved in study design, collection/analysis/interpretation of data or manuscript preparation, as was the Motor Neurone Disease Association.

R.L.G., M.A.S. and R.J.H. are supported by the NIHR University College London Hospitals Biomedical Research Centre at University College London Hospitals NHS Foundation Trust and University College London. M.Br., T.Y., C.C. and C.J.M. are supported by the NIHR Sheffield Biomedical Research Centre. A.A.‐C., V.L. and L.H.G. are supported by the NIHR Maudsley Biomedical Research Centre at South London and Maudsley NHS Foundation Trust and King's College London. C.J.M. is an NIHR Research Professor. A.A.‐C. is an NIHR Senior Investigator (NIHR202421), and is supported by an EU Joint Programme ‐ Neurodegenerative Disease Research (JPND) project through the following funding organisations under the aegis of JPND: www.jpnd.eu (United Kingdom, Medical Research Council [MR/L501529/1; MR/R024804/1] and Economic and Social Research Council [ES/L008238/1]). A.A.‐C. is also supported through the Motor Neurone Disease Association, My Name'5 Doddie Foundation and the Alan Davidson Foundation.

For the purposes of open access, the authors have applied a Creative Commons Attribution (CC BY) licence to any accepted author manuscript version arising from this submission.

## CONFLICT OF INTEREST STATEMENT

A.A.‐C. received consultancies from or sat on advisory boards for Therapeutics, Cytokinetics, GenieUs, GSK, Lilly, Mitsubishi Tanabe Pharma, Novartis, OrionPharma, Quralis, Sano, Sanofi and Wave Pharmaceuticals. None of the other authors have any conflicts of interest to declare.

## Supporting information


Appendix S1

## Data Availability

The data that support the findings of this study are available from the corresponding author upon reasonable request.
