# Supplementary material for: Cost‐effectiveness of acceptance and commitment therapy for people living with motor neuron disease, and their health‐related quality of life
Source: Eur J Neurol. 2024 Apr 25;31(8):e16317. doi: 10.1111/ene.16317 (PMC11235598; doi:10.1111/ene.16317)
Supplement: Supplementary file 1 — Appendix S1 [file ENE-31-e16317-s001.docx]

**SUPPLEMENTARY MATERIALS**

Contents

[**1.** **Health Economics Analysis Plan** 3](#_Toc151898271)

[**Deviations to protocol** 3](#_Toc151898272)

[**2.** **CHEERS checklist** 26](#_Toc151898273)

[**3.** **Long-term modelling** 29](#_Toc151898274)

[**Methods** 29](#_Toc151898275)

[Model structure 29](#_Toc151898276)

[Methods for identifying and estimating parameters 30](#_Toc151898277)

[Model uncertainty 30](#_Toc151898278)

[**Results** 31](#_Toc151898279)

[**4.** **Tables** 33](#_Toc151898280)

**LIST OF TABLES**

[**Table A1a Unit costs for Primary and Community Services** 33](#_Toc151926770)

[**Table A1b Unit costs for Hospital, nursing home or hospice inpatient services** 34](#_Toc151926771)

[**Table A1c Unit costs for Outpatient and day care services** 34](#_Toc151926772)

[**Table A1d Unit costs for Equipment** 35](#_Toc151926773)

[**Table A1e Unit costs for Home adaptations** 36](#_Toc151926774)

[**Table A1f Unit costs for Psychological Therapies** 36](#_Toc151926775)

[**Table A2 EQ-5D-5L Anxiety and depression domain (% of participants)** 36](#_Toc151926776)

[**Table A3 Mean resource use by treatment arm** 37](#_Toc151926777)

[**Table A4 Resource use** 38](#_Toc151926778)

[**Table A5a Mean resource use costs - (with complete data n = 134)** 40](#_Toc151926779)

[**Table A5b Mean resource use costs – charities and out of pocket (with complete data n = 134)** 40](#_Toc151926780)

[**Table A6a Mean resource use costs – NHS and local authorities (with MI)** 41](#_Toc151926781)

[**Table A6b Mean resource use costs – NHS and local authorities (with MI)** 41](#_Toc151926782)

[**Table A7 Intervention costs** 42](#_Toc151926783)

[**Table A8 Costs and QALYs (ITT with imputation)** 42](#_Toc151926784)

[**Table A9 Primary analysis ITT with MI** 42](#_Toc151926785)

[**Table A10 MQOL scores (ITT with imputation)^a^** 43](#_Toc151926786)

[**Table A11 Secondary analysis – cost-effectiveness results** 43](#_Toc151926787)

[**Table A12 Costs and QALYs (complete case)** 44](#_Toc151926788)

[**Table A13 Complete case analysis - cost-effectiveness results** 44](#_Toc151926789)

[**Table A14 Costs and QALYs (per protocol)** 45](#_Toc151926790)

[**Table A15 Per protocol analysis** 45](#_Toc151926791)

[**Table A16 Costs and QALYs (ITT societal perspective with multiple imputation)** 47](#_Toc151926792)

[**Table A17 ITT societal perspective with multiple imputation** 47](#_Toc151926793)

[**Table A18 Subgroups** 50](#_Toc151926794)

[**Table A19 Subgroup analysis based on ALSFRS-R severity** 51](#_Toc151926795)

[**Table A20 Subgroup analysis based on rates of deterioration** 51](#_Toc151926796)

**LIST OF FIGURES**

[**Figure A1 Confidence ellipse: Complete case analysis** 44](#_Toc151926682)

[**Figure A2 Cost-effectiveness acceptability curve: complete case analysis** 45](#_Toc151926683)

[**Figure A3 Confidence ellipse: Per protocol analysis** 46](#_Toc151926684)

[**Figure A4 Cost-effectiveness acceptability curve: per protocol analysis** 46](#_Toc151926685)

[**Figure A5 Confidence ellipse: Partial societal analysis** 47](#_Toc151926686)

[**Figure A6 Cost-effectiveness acceptability curve: Partial societal analysis** 48](#_Toc151926687)

[**Figure A7 Confidence ellipse: Subgroup analysis – minimal to mild severity** 48](#_Toc151926688)

[**Figure A8 Cost-effectiveness acceptability curve: Subgroup analysis – minimal to mild severity** 49](#_Toc151926689)

[**Figure A9 Confidence ellipse: Subgroup analysis – mild to moderate severity** 49](#_Toc151926690)

[**Figure A10 Cost-effectiveness acceptability curve: Subgroup analysis – mild to moderate severity** 50](#_Toc151926691)

[**Figure A11 Confidence ellipse: Subgroup analysis – lowest deterioration rate** 52](#_Toc151926692)

[**Figure A12 Cost-effectiveness acceptability curve: Subgroup analysis – lowest deterioration rate** 52](#_Toc151926693)

[**Figure A13 Confidence ellipse: Subgroup analysis – medium deterioration rate** 53](#_Toc151926694)

[**Figure A14 Cost-effectiveness acceptability curve: Subgroup analysis – medium deterioration rate** 53](#_Toc151926695)

[**Figure A15 Confidence ellipse: Subgroup analysis – highest deterioration rate** 54](#_Toc151926696)

[**Figure A16 Cost-effectiveness acceptability curve: Subgroup analysis – highest deterioration rate** 54](#_Toc151926697)

# **Health Economics Analysis Plan**

## **Deviations to protocol**

1. **In the HEAP, we specified that we would only use EQ-5D-5L as a measure of effectiveness. The intervention was found to be clinically effective but the economic evaluation showed that the intervention was not cost-effective. An additional analysis was added using the primary outcome of the trial as a measure of effectiveness.**
2. **For the long term modelling two deviations were made:**
3. **The HEAP stipulated that a**nalyses on EVPI and expected value of perfect partial information (EVPPI) would be undertaken. Given the very small and non-significant changes in QALYs in the 9-month **period, this was not undertaken.**
4. Deterministic sensitivity analysis was not undertaken given the within robust results of trial results.

# **Health Economics Analysis Plan**

| 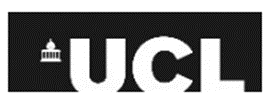 | 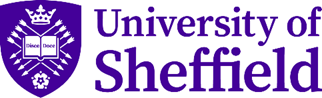 |
| --- | --- |

COMMEND

| **COMMEND Health Economic Analysis Plan v 1.0** |
| --- |

| Study Title | **A feasibility study and randomised controlled trial of Acceptance and Commitment Therapy for people with motor neuron disease (COMMEND)** |
| --- | --- |
| Funding body | **NIHR HTA** |
| Trial registration no: | **ISRCTN12655391** |

**Authors:** Professor Tracey Young and Dr Anju Keetharuth

ScHARR, University of Sheffield

# List of abbreviations used

| ACT | Acceptance and Commitment Therapy |
| --- | --- |
| ALS | Amyotrophic Lateral Sclerosis |
| ALS-FRS-R | Amyotrophic Lateral Sclerosis Functional Rating Scale- Revised questionnaire |
| CBT | Cognitive Behavioural Therapy |
| CEAC | Cost-effectiveness acceptability curve |
| EQ-5D-3L | EuroQoL 5 dimension – 3 level questionnaire |
| EQ-5D-5L | EuroQoL 5 dimension – 5 level questionnaire |
| HEAP | Health Economics Assessment Plan |
| ICER | Incremental cost-effectiveness ratio |
| ITT | Intention To Treat analysis |
| M-HADS | Hospital Anxiety and Depression Scale modified for people with MND |
| M-HADS-A | Modified Hospital Anxiety and Depression Scale – anxiety component |
| M-HADS-D | Modified Hospital Anxiety and Depression Scale – depression component |
| MQOL-R | McGill Quality of life –Revised |
| MND | Motor neuron disease |
| NIHR | National Institute for Health Research |
| PLS | Primary Lateral Sclerosis |
| PMA | Progressive Muscular Atrophy |
| PP | Per Protocol analysis |
| QALY | Quality Adjusted Life Year |
| RCT | Randomised controlled trial |
| SAP | Statistical analysis plan |
| TSC | Trial Steering Committee |

Table of Contents

[List of abbreviations used 2](#_Toc138908125)

[Section 1. Administrative Information 5](#_Toc138908126)

[Section 2. Trial Introduction & Background 5](#_Toc138908127)

[2.1 Trial Background & Rationale 5](#_Toc138908128)

[2.2 Aim of the Trial 6](#_Toc138908129)

[2.3 Objectives of the Trial 6](#_Toc138908130)

[2.4 Trial Design 6](#_Toc138908131)

[2.5 Trial Population 6](#_Toc138908132)

[2.6 Intervention and Comparators 7](#_Toc138908133)

[Section 3. Economic Approach 8](#_Toc138908134)

[3.1 Aims of economic evaluation 8](#_Toc138908135)

[3.2 Objectives of economic evaluation 8](#_Toc138908136)

[3.3 Overview of economic evaluation 8](#_Toc138908137)

[3.4 Jurisdiction 8](#_Toc138908138)

[3.5 Perspectives 8](#_Toc138908139)

[3.6 Time horizon 9](#_Toc138908140)

[Section 4. Economic Data Collection and Management 9](#_Toc138908141)

[4.1 Statistical software 9](#_Toc138908142)

[4.2 Identification of resources 9](#_Toc138908143)

[4.3 Measurement of resource use data 9](#_Toc138908144)

[4.4 Valuation of resource use data 9](#_Toc138908145)

[4.5 Identification of outcomes 10](#_Toc138908146)

[4.6 Measurement of outcomes 10](#_Toc138908147)

[4.7 Valuation of outcomes 10](#_Toc138908148)

[Section 5. Economic Data Analysis 10](#_Toc138908149)

[5.1 Analysis population 10](#_Toc138908150)

[5.2 Timing of analysis 11](#_Toc138908151)

[5.3 Discount rates for costs and benefits 11](#_Toc138908152)

[5.4 Cost-effectiveness thresholds 11](#_Toc138908153)

[5.5 Statistical decision rules 11](#_Toc138908154)

[5.6 Analysis of resource use 11](#_Toc138908155)

[5.7 Analysis of costs 11](#_Toc138908156)

[5.8 Analysis of outcomes 11](#_Toc138908157)

[5.9 Data cleaning for analysis 12](#_Toc138908158)

[5.10 Missing data 12](#_Toc138908159)

[5.11 Analysis of cost-effectiveness 12](#_Toc138908160)

[5.12 Sampling uncertainty 12](#_Toc138908161)

[5.13 Subgroup analysis of heterogeneity 13](#_Toc138908162)

[5.14 Sensitivity analysis 13](#_Toc138908163)

[Section 6. Modelling 14](#_Toc138908164)

[6.1 Decision analytic modelling 14](#_Toc138908165)

[6.2 Model type 14](#_Toc138908166)

[6.3 Model structure 14](#_Toc138908167)

[6.4 Treatment effect beyond the end of the trial 15](#_Toc138908168)

[6.5 Other key assumptions 15](#_Toc138908169)

[6.6 Methods for identifying and estimating parameters 15](#_Toc138908170)

[6.7 Model uncertainty 15](#_Toc138908171)

[6.8 Model validation 16](#_Toc138908172)

[Section 7. Reporting/Publishing 16](#_Toc138908173)

[7.1 Reporting Standards 16](#_Toc138908174)

[7.2 Reporting Deviations from the HEAP 16](#_Toc138908175)

[Section 8. Appendices 17](#_Toc138908176)

[Appendix 1 – Amendment History 17](#_Toc138908177)

[Section 9. References 18](#_Toc138908178)

# Section 1. Administrative Information

Table 1 HEAP Administrative Information

| Title | A feasibility study and randomised controlled trial of Acceptance and Commitment Therapy for people with motor neuron disease (COMMEND) |
| --- | --- |
| Trial registration number; registry | ISRCTN12655391 |
| Source of funding | NIHR HTA |
| Trial protocol version; date | This document has been written based on information contained in the trial protocol version 3.2 dated 23/09/2022 |
| Trial Statistical Analysis Plan (SAP) version, date | This document has been written based on information contained in the trial statistical analysis plan version 2 dated 30/01/2023. |
| Trial HEAP version | 1, 14/07/2023 |
| HEAP revisions |  |
| Roles and responsibilities | The HEAP was prepared by Tracey Young and Anju Keetharuth. The trial health economists are responsible for conducting and reporting the economic evaluation in accordance with the HEAP. |

# Section 2. Trial Introduction & Background

## 2.1 Trial Background & Rationale

The current prognosis for motor neuron disease (MND) is poor with median survival of 2-3 years following onset and only 4-10% of people with MND surviving for more than 10 years [Al-Chalabi et al 2013; Chio et al 2009; Turner et al 2003]. Prevalence rates for depression and anxiety are high in people with MND (44% for depression, 30% for anxiety) and MND has been found to be the most frequent cause of assisted suicide [Averill et al, 2007; Kurt et al 2007; Taylor et al 2010]. Typically, formal psychotherapies such as Cognitive Behavioural Therapy (CBT) cannot meet the specific needs of people with MND in a timely fashion.

A systematic review [Gould et al, 2015] identified four studies that evaluated psychotherapies for people with MND [Averill et al, 2013; Diaz et al, 2016; Bentley et al 2014; Aoun et al 2015]. These studies were based on small samples and although some showed short-term improvements in well-being none showed evidence of maintaining this at follow-up. Further no studies looked at the cost-effectiveness of psychotherapy. Acceptance and Commitment Therapy (ACT) is an alternative form of psychological therapy to traditional therapies such as CBT. ACT focuses on increasing personally meaningful behaviour in the presence of distress and symptoms and has been applied to a wide range of health conditions with beneficial effects being reported [Hann et al, 2014; Graham et al 2016; McCracken et al, 2014; Sharp et al 2012; Smout et al 2012]. To date ACT has not yet been applied to people with MND.

## 2.2 Aim of the Trial

To determine the clinical and cost effectiveness of ACT, modified for people with MND, plus usual
multidisciplinary care in comparison to usual multidisciplinary care alone for improving psychological health in people with MND.

## 2.3 Objectives of the Trial

1. To establish the clinical and cost effectiveness of ACT plus usual multidisciplinary care for people with MND compared to usual multidisciplinary care alone, via a randomised controlled trial (RCT) with an internal pilot phase.
2. To evaluate the effect of ACT plus usual multidisciplinary care for people with MND compared to usual multidisciplinary care alone on caregivers of people with MND.
3. To examine perceived mechanisms of impact and the context in which the intervention is delivered by collecting qualitative data from people with MND and study therapists.

## 2.4 Trial Design

COMMEND is a two-arm parallel pragmatic RCT which investigates the effectiveness of ACT for people diagnosed with MND [Gould et al, 2022]. The expected duration of the COMMEND trial is 29 months, with a 10-month internal pilot to examine the feasibility of recruitment and acceptability of ACT. Participants are from at least 14 sites across the UK and are randomised into ACT along with usual multidisciplinary care or care alone in a 1:1 ratio.

## 2.5 Trial Population

***Inclusion criteria for participants with MND:***

1. Aged 18 years and over.
2. Diagnosis of definite, laboratory-supported probable, clinically probable, or possible familial or sporadic Amyotrophic lateral sclerosis (ALS) (which is diagnostically synonymous with MND) [Al-Chalabi et al 2016] using the World Federation of Neurology’s El Escorial criteria [Brooks et al 2000], and additionally the Progressive Muscular Atrophy (PMA) and Primary Lateral Sclerosis (PLS) variants where appropriate investigation has excluded mimics of MND.

It should be noted that all people with MND will be eligible to participate. There is no minimum threshold for anxiety or depression: people who do not have these symptoms are still eligible, but presence of severe psychiatric disorder (such as schizophrenia or bipolar disorder) is an exclusion criterion (see point 6 below).

***Inclusion criteria for caregivers:***

1. Aged 18 years and over.
2. Primary informal caregiver of a person with MND who has consented to participate in the trial.

***Inclusion criteria for study therapists:***

1. Aged 18 years and over.
2. Therapists who are involved in delivering the intervention to people with MND in the trial.

***Exclusion criteria***

1. Need for any form of gastrostomy feeding or non-invasive ventilation, as defined by a current clinical need. A clinical need is defined as the participant being dependent upon percutaneous endoscopic gastronomy to meet all their nutrition and hydration needs or meeting the NICE criteria for the offer of a trial of non-invasive ventilation as defined in section 1.14.17 of NICE Guidance NG42 (2011).
2. Diagnosis of dementia using standard diagnostic guidelines.
3. Currently receiving ongoing formal psychological therapy delivered by a formally trained psychologist or psychotherapist and unwilling to refrain from engaging in such formal psychological therapy during the receipt of ACT.
4. Insufficient understanding of English to enable engagement in ACT and completion of screening measures and patient-reported outcome measures.
5. Lacking capacity to provide fully informed written consent, verbal consent (for those who cannot provide written consent), or consent via the use of a communication aid.
6. Need for treatment for severe psychiatric disorder such as schizophrenia or bipolar disorder or expressing suicidal ideation with active plans/suicidal behaviours and imminent intent (hereafter defined as reports of plans to end one's life within the next 2 weeks).
7. Other medical factors that could compromise full study participation such as intellectual disabilities or severe sensory deficits (e.g., visual blindness).
8. Previous participation in Phase 1 of COMMEND (feasibility study).

## 2.6 Intervention and Comparators

Intervention: ACT plus usual multidisciplinary care for people with MND

Comparator: usual multidisciplinary care only for people with MND

# Section 3. Economic Approach

## 3.1 Aims of economic evaluation

The health economic analysis aims to assess the cost-effectiveness of ACT plus usual multidisciplinary care for people with MND, over the study period and modelled to lifetime using a cost per quality adjusted life-year (QALY) approach.

## 3.2 Objectives of economic evaluation

The primary objective of the health economic evaluation is to estimate the cost-effectiveness of ACT with multidisciplinary care over the timeframe of the study for people with MND. A secondary objective will be to estimate the long-term cost-effectiveness modelled to lifetime using a cost per quality adjusted life-year (QALY) approach.

3.3 Overview of economic evaluation

The within trial analysis will be performed using individual level data from the COMMEND trial. The analytical approaches will take the form of cost-utility analyses. Based on trial evidence, incremental cost-effectiveness ratios (ICER) will be calculated by taking a ratio of the difference in the mean costs and mean QALYs using the utilities from the preference-based measure.

A Markov model will be constructed to explore the cost-effectiveness of ACT with multidisciplinary care over the life course of the disease. This five-state model will use the Kings’ staging [Fang et al 2017] and a one-month cycle (more details in Section 6). As with the trial-based analysis, results will be presented in terms of an ICER.

3.4 Jurisdiction

The trial will be conducted in the UK setting, which has a national health service (NHS), providing publicly funded healthcare, primarily free of charge at the point of use.

## 3.5 Perspectives

The primary analysis will take an NHS and Social Care perspective. A secondary analysis will consider a wider perspective that will include the costs incurred by caregivers.

## 3.6 Time horizon

The primary economic analysis will compare the costs and QALYs over the study period (9 months). A Markov model will then be used to estimate the cost-effectiveness over the lifetime following randomisation.

# Section 4. Economic Data Collection and Management

## 4.1 Statistical software

R studio version 2023.06.0 or higher and R version 4.2.2 or later (R Core Team) and Stata version 17 or higher (Stata Corp) will be used for cost-effectiveness analysis.

## 4.2 Identification of resources

The costing approach will include identification of resource use, measurement and valuation. Resource use data will be collected as part of the trial at baseline (asking participants to recollect their use for the last six months), 6- and 9-months (asking participants to recollect their use for the last three months) follow-up using a modified version of the Client Service Receipt Inventory [Beecham & Knapp, 1992]. In addition, staff training and delivery of the intervention will be measured to assess the cost of the intervention.

## 4.3 Measurement of resource use data

Relevant health care resource use captured during the trial will include information on hospital, nursing home and hospice services, out-patient visits and day care, primary and secondary community care services, equipment obtained and home adaptations. Resource use information will be collected until 9 months post randomisation using trial case report forms and patient questionnaires. The resource use will be reported for the whole period but also separately to reflect the possible effects of the various stages of the pandemic (prior to 23^rd^ March 2020; between 23^rd^ March 2020 and 27^th^ Jan 2022; 27^th^ Jan 2022 onwards).

## 4.4 Valuation of resource use data

Each of the resource use items will be valued in monetary terms, where costs are not provided by the participants, appropriate unit costs from standard health economic sources such as

- British National Formulary (Joint Formulary Committee)
- NHS Reference costs (Department of Health)
- PSSRU Unit costs of health and social care [Jones et al 2022]
- NHS Agenda for Change (2022)
- Office of National Statistics annual survey of hours and earnings (2022).

Costs are calculated using Great British Pounds (GBP) for the year of analysis; where costs are not available for this year, they will be inflated using the hospital and community health services (HCHS) index [Jones et al. 2022].

## 4.5 Identification of outcomes

The primary economic evaluation outcome measure will be QALYs derived from the EQ-5D-5L quality of life instrument.

## 4.6 Measurement of outcomes

Measurements will be recorded prior to randomisation at baseline and at 6- and 9-months. Participants are asked about their resource use for the last 6 months at baseline and 6-months visits, and for the last 3 months at the 9-months data collection point.

## 4.7 Valuation of outcomes

Utility scores will be derived from responses to the EQ-5D-5L. UK utility values will be derived using the mapping function from the EQ-5D-3L, which is the approach recommended by NICE [NICE, 2022]. These will be used to estimate QALYs over the 9-month study period. A utility score of 0 will be assigned on date of death for the purpose of QALY computation.

# Section 5. Economic Data Analysis

## 5.1 Analysis population

The primary analysis set will include all randomised participants, which is in accordance with the “intention to treat” (ITT) principle. A secondary analysis will be conducted on the per-protocol population as defined in section 5.14.

## 5.2 Timing of analysis

The primary analysis will be conducted once all participants have completed their final follow-up. The final analysis will include the within-trial analysis and model-based analysis.

## 5.3 Discount rates for costs and benefits

Costs and QALYs will be discounted at 3.5% per annum as recommended by NICE for the lifetime model [NICE, 2022]. Discounting will not be relevant for the trial-based analysis.

## 5.4 Cost-effectiveness thresholds

A range of willingness to pay thresholds will be considered for the cost-utility analyses, including the NICE threshold of £20,000 per QALY, as per NICE guidance [NICE, 2022].

## 5.5 Statistical decision rules

Mean differences in costs, QALYs, and ICER will be estimated with associated 95% confidence intervals.

## 5.6 Analysis of resource use

Differences in the use of services between randomised groups will be described but not compared statistically.

## 5.7 Analysis of costs

Histograms of total costs for complete case data will be used to visualise the distribution of cost data. Mean total costs in each arm will be summarised and then broken down into the different components of resource use to identify the drivers in the total costs.

## 5.8 Analysis of outcomes

Multiple regression model will be used to adjust for any imbalance in baseline utility across the arms. Mean QALY at each time point will be plotted visually using a line plot to illustrate the pattern of utility over the trial period for each intervention. Mean QALY will be calculated using the area under the curve method. If the costs and outcomes are correlated, this will be taken into consideration by using seemingly unrelated regressions.

## 5.9 Data cleaning for analysis

Data will be checked for face validity and any unusual results queried with the study team. Any changes to the data will be documented and implemented using R/Stata code. No changes will be made to the original data.

## 5.10 Missing data

Missing data can give misleading estimates of a within-trial cost-effectiveness analysis. A complete-case analysis uses only participants with no missing data in the key cost and benefit outcomes. This is undesirable as it reduces the sample size and affects the power of the study [Faria et al 2014]. If required, patterns of missing data will be assessed using the approach outlined by [Faria et al 2014] and will include a descriptive analysis of:

1. Proportion of missing data by treatment arm, at each follow-up period, to assess whether missing data differed by arm.
2. Missing data patterns to determine whether data were missing for all items or individual items of utility scores and resource use items over the trial follow-up.
3. Proportion of survival at 9 months by arm

If deemed to be appropriate multiple imputation will be used to impute missing values. The number of imputations will be based on the highest percentage of missing data for the variables of interest. The imputation will be performed per randomisation arm, for all imputed variables, except baseline covariates with missing data, for which imputation will be performed across all observations.

## 5.11 Analysis of cost-effectiveness

Cost-utility analysis (CUA) will be performed to compare the cost-effectiveness of ACT with multidisciplinary care compared to usual multidisciplinary care alone. Seemingly Unrelated Regression (SUR) will be used, if appropriate, to account for the correlation between costs and QALYs. Cost and QALY data will be combined to calculate an incremental cost-effectiveness ration (ICER) and net monetary benefit statistic from the NHS and social health care prospective.

## 5.12 Sampling uncertainty

The value threshold per unit of effectiveness will be varied to assess the uncertainty associated with the estimates. Additionally, a cost-effectiveness acceptability curve (CEAC) will be constructed illustrating the probability of each treatment being most cost-effective for a range of threshold values.

## 5.13 Subgroup analysis of heterogeneity

Analysis will be conducted on the final dataset to investigate how cost-effectiveness varies between different patient subgroups. Heterogeneity will be explored if there is a statistically significant difference in the primary outcome, the McGill Quality of Life-Revised (MQOL-R). A p value of 0.1 instead of the usual p = 0.05 will be used in line with guidance from the European Medicines Agency [EMEA 1998] Furthermore, heterogeneity will be explored between subgroups and for subgroups of 50 participants or more. Subgroups that will be considered include: severity of condition at baseline using ALS-FRS-R, severity of baseline depression and anxiety using Modified Hospital Anxiety and Depression Scale – depression component (M-HADS-D) and Modified Hospital Anxiety and Depression Scale – anxiety component (M-HADS-A), rate of deterioration and participants recruited during the COVID-19 pandemic versus those recruited pre and post the restrictions. We will use 3 subgroups with cut points for before and after the beginning of national lockdown on 23 March 2020 and before and after the final easing of lockdown restrictions on 27 January 2022. Separate subgroups between these dates will not be used because although the rules changed frequently during this period people did not begin to return to usual behaviours until the final easing on 27 January 2022), (See SAP Section 7.7 for further details on subgroups).

## 5.14 Sensitivity analysis

Several sensitivity analyses including a probabilistic sensitivity analysis, will be performed to assess the robustness of the within-trial health economic estimates. Subgroup analyses will be reported if appropriate. The analyses include:

- An analysis taking into account the societal perspective including caregiver costs and different choice of threshold
- A complete case analysis will be carried out to include all randomised participants that have complete data on cost and effectiveness outcomes.
- A PP analysis set will include all participants who received at least 4 sessions within a four-month period and prior to the 6-month follow-up visit

# Section 6. Modelling

## 6.1 Decision analytic modelling

A model-based approach will be used to extrapolate the analysis beyond the trial follow-up to assess whether there is potential for the cost -effectiveness of the intervention to improve under a longer analysis time horizon than the 9 months follow-up.

## 6.2 Model type

A cohort-state transition model will be used to evaluate effects of the intervention on costs, health gains and cost-effectiveness over the remaining lifetime of people with MND. The cycle length (the duration of each interval in which changes in patients’ health state and consequent impacts on mortality risk, health-related quality of life (HRQoL) and costs are evaluated) is one month.

## 6.3 Model structure

A mean change in EQ-5D-5L will be calculated from the trial data. The increment to baseline EQ-5D-5L score will be added to an existing model [Tappenden 2023] with 5 stages based on the King’s staging classification. In this trial, disease severity is measured using the ALS-Functional Rating Scale- Revised (ALSFRS-R) and the model uses King’s staging classification which is not based on ALSFRS-R. However, the King’s staging system can be estimated with 92% concordance [Balendra 2015]. We will use an existing algorithm which maps ALSFRS-R onto EQ-5D in people with MND [Moore 2018]. The model structure is shown in Figure 1.

Figure 1 Markov model structure based on the King’s staging classification


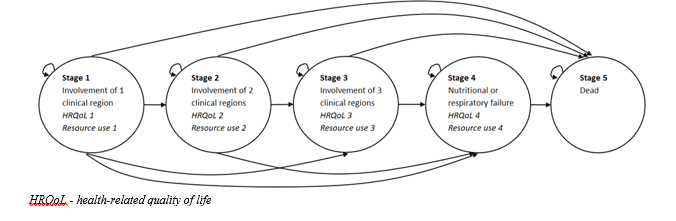


## 6.4 Treatment effect beyond the end of the trial

Two scenarios will be tested. First, it is assumed that any psychological benefit experienced will be sustained beyond the end of the trial and the increased QALY associated with psychological benefit will persist for as long the person stays alive. Second, it is assumed that the psychological benefit lasts for the duration of the intervention only. These two scenarios provide the most optimistic and pessimistic scenarios. These two scenarios may be replaced with a more realistic scenario if additional evidence on the effects of ACT for people with chronic muscle diseases emerges at the time of analysis (Rose 2022). Evidence in the latter population is the closest available evidence in the absence of evidence of ACT in people with MND.

## 6.5 Other key assumptions

The following assumptions will be made:

- All patients enter the model in Stage 2. This assumption was made by Tappenden et al [2023], based on clinical opinion and it will be tested based on baseline staging data and necessary modification will be made as appropriate.
- During any model cycle, from any alive state, patients can only skip stages forward (patients do not get to a less severe state).

## 6.6 Methods for identifying and estimating parameters

The model requires 4 main set of parameters: a. transition probabilities between health states b. treatment effects of the intervention group c. utility decrements d. costs. The transition probabilities from Tappenden et al [2023) will be used. We will assume that the treatment response occurs at month 2 given the duration of the intervention. Costs will be obtained from the trial and other published sources. The utilities at the end of the trial period will be extrapolated for the whole lifetime by using established methods for age adjustments.

## 6.7 Model uncertainty

A probabilistic sensitivity analysis (PSA) will be undertaken and the distributions for the model parameters will be chosen based on within trial analysis. Assumptions will be made in assigning different distributions. A discount rate of 3.5% will be used based on NICE reference case. The PSA will be run for 10,000 iterations to address uncertainty associated with the parameters estimates used to populate the model. Analyses on EVPI and expected value of perfect partial information (EVPPI) will be undertaken. Deterministic sensitivity analysis will also be undertaken (e.g. varying costs and utilities) and results will be presented using tables and appropriate diagrams.

## 6.8 Model validation

The model will be estimated and internally validated using trial data.

# Section 7. Reporting/Publishing

## 7.1 Reporting Standards

The results of the within trial analysis will be reported in line with the CHEERS checklist for reporting economic evaluations [Husereau 2022]. This HEAP was written using the template provided by Thorn et al [2021].

## 7.2 Reporting Deviations from the HEAP

Any deviation from the HEAP will be described and justified in the final published report.

# Section 8. Appendices

## Appendix 1 – Amendment History

| **Amendment No.** | **HEAP version no.** | **Date issued** | **Author(s) of changes** | **Details of changes made** |
| --- | --- | --- | --- | --- |
|  |  |  |  |  |

# Section 9. References

Al-Chalabi A, Hardiman O. The epidemiology of ALS: a conspiracy of genes, environment and time. Nature Reviews Neurology Rev Neurol [Internet]. 2013 Nov 15 [cited 2018 Sep 20];9(11):617–28. Available from: http://www.ncbi.nlm.nih.gov/pubmed/24126629

Al-Chalabi A, Hardiman O, Kiernan MC, Chiò A, Rix-Brooks B, van den Berg LH. Amyotrophic lateral sclerosis: moving towards a new classification system. The Lancet Neurology. 2016 Oct 1;15(11):1182-94. [https://doi.org/10.1016/S1474-4422(16)30199-5](about:blank)

Aoun SM, Chochinov HM, Kristjanson LJ. Dignity Therapy for People with Motor Neuron Disease and Their Family Caregivers: A Feasibility Study. Journal of Palliative Medicine [Internet]. 2015 Jan [cited 2018 Sep 21];18(1):31–7. Available from: [http://www.ncbi.nlm.nih.gov/pubmed/25314244](about:blank)

Averill AJ, Kasarskis EJ, Segerstrom SC. Psychological health in patients with amyotrophic lateral sclerosis. Amyotrophic Lateral Sclerosis [Internet]. 2007 Jan 10 [cited 2018 Sep 20];8(4):243–54. Available from: [http://www.ncbi.nlm.nih.gov/pubmed/17653923](about:blank)

Averill AJ, Kasarskis EJ, Segerstrom SC. Expressive disclosure to improve well-being in patients with amyotrophic lateral sclerosis: A randomised, controlled trial. Psychology & Health [Internet]. 2013 Jun [cited 2018 Sep 21];28(6):701–13. Available from: [http://www.ncbi.nlm.nih.gov/pubmed/23289543](about:blank)

Balendra, R., Jones, A., Jivraj, N., Steen, I.N., Young, C.A., Shaw, P.J., Turner, M.R., Leigh, P.N., Al-Chalabi, A., UK-MND LiCALS Study Group and Mito Target ALS Study Group, 2015. Use of clinical staging in amyotrophic lateral sclerosis for phase 3 clinical trials. Journal of Neurology, Neurosurgery & Psychiatry, 86(1), pp.45-49.

Beecham J, Knapp M. Costing psychiatric interventions. In G. Thornicroft, C. R. Brewin, & J. Wing (Eds.), Measuring mental health needs (pp. 163–183). Gaskell/Royal College of Psychiatrists.

Bentley B, O’Connor M, Breen LJ, Kane R. Feasibility, acceptability and potential effectiveness of dignity therapy for family carers of people with motor neurone disease. BMC Palliative Care [Internet]. 2014 Dec 19 [cited 2018 Sep 21];13(1):12. Available from: [http://bmcpalliatcare.biomedcentral.com/articles/10.1186/1472-684X-13-12](about:blank)

Brooks BR, Miller RG, Swash M, Munsat TL. El Escorial revisited: revised criteria for the diagnosis of amyotrophic lateral sclerosis. Amyotrophic Lateral Sclerosis and other Motor Neuron Disorders. 2000 Jan 1;1(5):293-9.

Chiò A, Logroscino G, Hardiman O, Swingler R, Mitchell D, Beghi E, et al. Prognostic factors in ALS: A critical review. Amyotrophic Lateral Sclerosis [Internet]. 2009 Jan 18 [cited 2018 Sep 20];10(5–6):310–23. Available from: [http://www.ncbi.nlm.nih.gov/pubmed/19922118](about:blank)

Department of Health. Reference Costs 2019/20. [Internet]. 2021. Available from: [https://digital.nhs.uk/data-and-information/data-collections-and-data-sets/data-collections/reference-costs](about:blank)

Díaz JL, Sancho J, Barreto P, Bañuls P, Renovell M, Servera E. Effect of a short-term psychological intervention on the anxiety and depression of amyotrophic lateral sclerosis patients. Journal of Health Psychology [Internet]. 2016 Jul 4 [cited 2018 Sep 21];21(7):1426–35. Available from: [http://journals.sagepub.com/doi/10.1177/1359105314554819](about:blank)

European Medicines Agency (EMEA). Note for guidance on statistical principles for clinical trials. Statistical Principles for Clinical Trials ICH Topic E9. Available from: [E 9 Statistical Principles for Clinical Trials (europa.eu)](https://www.ema.europa.eu/en/documents/scientific-guideline/ich-e-9-statistical-principles-clinical-trials-step-5_en.pdf)

Fang T, Al Khleifat A, Stahl DR, Lazo La Torre C, Murphy C, Uk-Mnd LicalS, Young C, Shaw PJ, Leigh PN, Al-Chalabi A. Comparison of the King’s and MiToS staging systems for ALS. Amyotrophic Lateral Sclerosis and Frontotemporal Degeneration. 2017 Apr 3;18(3-4):227-32.

Faria R, Gomes M, Epstein D, White IR. A guide to handling missing data in cost-effectiveness analysis conducted within randomised controlled trials. Pharmacoeconomics. 2014;32(12):1157–70.

Gould RL, Coulson MC, Brown RG, Goldstein LH, Al-Chalabi A, Howard RJ. Psychotherapy and pharmacotherapy interventions to reduce distress or improve well-being in people with amyotrophic lateral sclerosis: A systematic review. Amyotrophic Lateral Sclerosis Frontotemporal Degeneration [Internet]. 2015 Aug 27 [cited 2018 Sep 21];16(5–6):293–302. Available from: [http://www.ncbi.nlm.nih.gov/pubmed/26174444](about:blank)

Gould, R.L., Thompson, B.J., Rawlinson, C., Kumar, P., White, D., Serfaty, M.A., Graham, C.D., McCracken, L.M., Bursnall, M., Bradburn, M., Young, T., Howard, R.J., Al-Chalabi, A., Goldstein, L.H., Lawrence, V., Cooper, C., Shaw, P., McDermott, C.J. (2022). A randomised controlled trial of Acceptance and Commitment Therapy plus usual care compared to usual care alone for improving psychological health in people with motor neuron disease (COMMEND): Study protocol. *BMC Neurology,* 22:431*.* doi: 10.1186/s12883-022-02950-5.

Graham CD, Gouick J, Krahé C, Gillanders D. A systematic review of the use of Acceptance and Commitment Therapy (ACT) in chronic disease and long-term conditions. Clinical Psychology Review [Internet]. 2016 Jun [cited 2018 Sep 20];46:46–58. Available from: [http://www.ncbi.nlm.nih.gov/pubmed/27176925](about:blank)

Hann KEJ, McCracken LM. A systematic review of randomized controlled trials of Acceptance and Commitment Therapy for adults with chronic pain: Outcome domains, design quality, and efficacy. Journal of Contextual Behavioural Science [Internet]. 2014 Oct 1 [cited 2018 Sep 20];3(4):217–27. Available from: [https://www.sciencedirect.com/science/article/pii/S2212144714000787](about:blank)

Husereau D, Drummond M, Augustovski F, de Bekker-Grob E, Briggs AH, Carswell C, et al. Consolidated health economic evaluation reporting standards 2022 (CHEERS 2022) statement: Updated Reporting Guidance for Economic Evaluations. Value in Health. 2022;25(1):3-9. PMID: [35031096](about:blank)

Joint Formulary Committee. British National Formulary (online) [Internet]. Available from: [http://www.medicinescomplete.com](about:blank)

Jones, Karen, Weatherly, Helen Louise Ann , Birch, Sarah et al. (2022) Unit Costs of Health and Social Care 2022. Report. Personal Social Services Research Unit, University of Kent at Canterbury , Kent. Available from: https://www.pssru.ac.uk/unitcostsreport/

Kurt A, Nijboer F, Matuz T, Kübler A. Depression and anxiety in individuals with amyotrophic lateral sclerosis: epidemiology and management. CNS Drugs [Internet]. 2007 [cited 2018 Sep 20];21(4):279–91. Available from: [http://www.ncbi.nlm.nih.gov/pubmed/17381183](about:blank)

McCracken LM, Vowles KE. Acceptance and commitment therapy and mindfulness for chronic pain: Model, process, and progress. Am Psychol [Internet]. 2014 [cited 2018 Sep 21];69(2):178–87. Available from: [http://www.ncbi.nlm.nih.gov/pubmed/24547803](about:blank)

Moore A, Young CA, Hughes DA. Economic studies in motor neurone disease: a systematic methodological review. Pharmacoeconomics. 2017 Apr;35:397-413.

Moore A, Young CA, Hughes DA. Mapping ALSFRS-R and ALSUI to EQ-5D in Patients with Motor Neuron Disease. Value Health. 2018 Nov;21(11):1322-1329. doi: 10.1016/j.jval.2018.05.005.

National Institute for Health and Care Excellence. Motor neurone disease: Assessment and management (NG42). [Internet]. 2011. Available from: [www.nice.org.uk/Guidance/NG42](http://www.nice.org.uk/Guidance/NG42).

National Institute for Health and Care Excellence. Guide to the Methods of Technology Appraisal [Internet]. 2022 [cited 2023 Jan 06]. Available from: [https://www.nice.org.uk/process/pmg36/chapter/introduction-to-health-technology-evaluation](about:blank)

Palmieri A, Kleinbub JR, Calvo V, Sorarù G, Grasso I, Messina I, et al. Efficacy of Hypnosis-Based Treatment in Amyotrophic Lateral Sclerosis: A Pilot Study. Frontiers in Psychology [Internet]. 2012 [cited 2018 Sep 21];3:465. Available from: [http://www.ncbi.nlm.nih.gov/pubmed/23162510](about:blank)

R Core Team. R: A language and environment for statistical computing. R Foundation for Statistical Computing [Internet]. Vienna, Austria; 2018. Available from: [https://www.r-project.org/](about:blank)

Rose, M., Graham, C.D., O'Connell, N., Vari, C., Edwards, V., Taylor, E., McCracken, L.M., Radunovic, A., Rakowicz, W., Norton, S. and Chalder, T., 2022. A randomised controlled trial of acceptance and commitment therapy for improving quality of life in people with muscle diseases. Psychological medicine, pp.1-14.

Sharp K. A Review of Acceptance and Commitment Therapy with Anxiety Disorders [Internet]. Vol. 12, International Journal of Psychology & Psychological Therapy. 2012 [cited 2018 Sep 21].

Smout MF, Hayes L, Atkins PWB, Klausen J, Duguid JE. The empirically supported status of acceptance and commitment therapy: An update. Clinical Psychologist [Internet]. 2012 Nov 1 [cited 2018 Sep 21];16(3):97–109. Available from: [http://doi.wiley.com/10.1111/j.1742-9552.2012.00051.x](about:blank)

StataCorp. Stata Statistical Software: Release 15. College Station, TX: StataCorp LLC; 2017.

Tappenden P., Navega Biz A., Ren S., McDermott C. (2023). Development of a baseline health economic model to provide a platform for assessing the cost-effectivebness of future treatments for amyotrophc lateral sclerosis (ALS). Final report to the EU Joint Programme on Neurodegenerative Disease (JPND) Research. Unpublished.

Taylor L, Wicks P, Leigh PN, Goldstein LH. Prevalence of depression in amyotrophic lateral sclerosis and other motor disorders. European Journal of Neurology [Internet]. 2010 Aug [cited 2018 Sep 20];17(8):1047–53. Available from: [http://www.ncbi.nlm.nih.gov/pubmed/20158515](about:blank)

Thorn J, Davies C, Brookes S, Noble S, Dritsaki M, Gray E, et al. Content of Health Economics Analysis Plans (HEAPs) for Trial-Based Economic Evaluations: Expert Delphi Consensus Survey. Value in Health. 2021 Apr 1;24(4):539-47

Turner MR, Parton MJ, Shaw CE, Leigh PN, Al-Chalabi A. Prolonged survival in motor neuron disease: a descriptive study of the King’s database 1990-2002. Journal of Neurology, Neurosurgery & Psychiatry [Internet]. 2003 Jul [cited 2018 Sep 20];74(7):995–7. Available from: [http://www.ncbi.nlm.nih.gov/pubmed/12810805](about:blank)

# **CHEERS checklist**

**Re:** Husereau D, Drummond M, Augustovski F, et al. Consolidated Health Economic Evaluation Reporting Standards 2022 (CHEERS 2022) statement: updated reporting guidance for health economic evaluations. *International journal of technology assessment in health care*. 2022;38(1):e13.

| **Section/Topic** | **Item No** | **Guidance for reporting** | **Reported in Section** |
| --- | --- | --- | --- |
| *Title* | | | |
| Title | 1 | Identify the study as an economic evaluation and specify the interventions being compared. | Title page |
| *Abstract* | | | |
| Abstract | 2 | Provide a structured summary that highlights context, key methods, results, and alternative analyses. | Abstract |
| *Introduction* | | | |
| Background and objectives | 3 | Give the context for the study, the study question, and its practical relevance for decision making in policy or practice. | Background |
| *Methods* | | | |
| Health economic  analysis plan | 4 | Indicate whether a health economic analysis plan was developed and where available. | Methods and HEAP available in Supplementary materials 1 |
| Study population | 5 | Describe characteristics of the study population (such as age range, demographics, socioeconomic or clinical characteristics) | Methods - study design and participants |
| Setting and location | 6 | Provide relevant contextual information that may influence findings | Methods - study design and participants |
| Comparators | 7 | Describe the interventions or strategies being compared and why chosen | Methods - the intervention |
| Perspective | 8 | State the perspective(s) adopted by the study and why chosen | Methods - Type of evaluation, perspective, and length of study |
| Time horizon | 9 | State the horizon for the study and why appropriate | Methods - Type of evaluation, perspective, and length of study |
| Discount rate | 10 | Report the discount rate(s) and why chosen | No discounting as trial period is 9 months |
| Selection of outcomes | 11 | Describe what outcomes were used as the measure(s) of beneﬁt(s) and harm(s). | Methods - Outcomes |
| Measurement of outcomes | 12 | Describe how outcomes used to capture beneﬁt(s) and harm(s) were measured. | Methods - Outcomes |
| Valuation of outcomes | 13 | Describe the population and methods used to measure and value outcomes | Methods - Outcomes |
| Measurement and valuation of resources and costs | 14 | Describe how costs were valued | Methods - resource use and unit costs |
| Currency, price, date and conversion | 15 | Report the dates of the estimated resource quantities and unit costs plus the currency and year of conversion | Methods - resource use and unit costs |
| Rationale and description of model | 16 | If modelling is used, describe in detail and why used. Report if the model is publicly available and where it can be accessed. | Supplementary Materials 3 |
| Analytics and assumptions | 17 | Describe any methods for analysing or statistically transforming data, any extrapolation methods, and approaches for validating any model used. | Methods - statistical analysis |
| Characterizing heterogeneity | 18 | Describe any methods used for estimating how the results of the study vary for subgroups. | Methods - Sensitivity and subgroup |
| Characterizing distributional effects | 19 | Describe how impacts are distributed across different individuals or adjustments made to reﬂect priority populations. | Not applicable |
| Characterizing uncertainty | 20 | Describe methods to characterise any sources of uncertainty in the analysis. | Methods - Statistical analysis; Sensitivity and subgroup |
| Approach to engagement with patients and others affected by the study | 21 | Describe any approaches to engage patients or service recipients, the general public, communities, or stakeholders (such as clinicians or payers) in the design of the study. | Not applicable - reported elsewhere |
| *Results* | | | |
| Study parameters | 22 | Report all analytic inputs (such as values, ranges, references) including uncertainty or distributional assumptions. | Supplementary Materials 3 |
| Summary of main results | 23 | Report the mean values for the main categories of costs and outcomes of interest and summarise them in the most appropriate overall measure | Results - various sections |
| Effect of uncertainty | 24 | Describe how uncertainty about analytic judgments, inputs, or projections affect ﬁndings.  Report the effect of choice of discount rate and time horizon, if applicable. | Not applicable |
| Effect of engagement with patients and others affected by the study | 25 | Report on any difference patient/service recipient, general public, community, or stakeholder involvement made to the approach or  ﬁndings of the study | Not applicable - reported elsewhere |
| *Discussion* | | | |
| Study ﬁndings, limitations, generalizability, and current knowledge | 26 | Report key ﬁndings, limitations, ethical or equity considerations not captured, and how these could affect patients, policy, or practice. | Discussion |
| *Other relevant information* | | | |
| Source of funding | 27 | Describe how the study was funded and any role of the funder in the identiﬁcation, design, conduct, and reporting of the analysis | Funding statement |
| Conflicts of interest | 28 | Report authors conflicts of interest according to journal or International committee of Medical Journal Editors requirements | Conflict of interest statement |

# **Long-term modelling**

## **Methods**

A model-based approach was used to extrapolate the analysis beyond the trial follow-up to assess whether there is potential for the cost -effectiveness of the intervention to improve under a longer analysis time horizon than the 9 months follow-up. A cohort-state transition model was used to evaluate effects of the intervention on costs, health gains and cost-effectiveness over the remaining lifetime of people with MND. The cycle length (the duration of each interval in which changes in patients’ health state and consequent impacts on mortality risk, health-related quality of life (HRQoL) and costs are evaluated) is one month.

### Model structure

The mean change in EQ-5D-5L from the primary within-trial analysis was added to an existing model [Tappenden 2023] with 5 stages based on the King’s staging classification. In this trial, disease severity is measured using the ALS-Functional Rating Scale- Revised (ALSFRS-R) and the model uses King’s staging classification which is not based on ALSFRS-R. However, the King’s staging system can be estimated with 92% concordance [Balendra 2015]. We used an existing algorithm which maps ALSFRS-R onto EQ-5D-5L in people with MND [Balendra 2018]. The model structure is shown in Figure 1.

Figure 1 Markov model structure based on the King’s staging classification

Long-term model structure


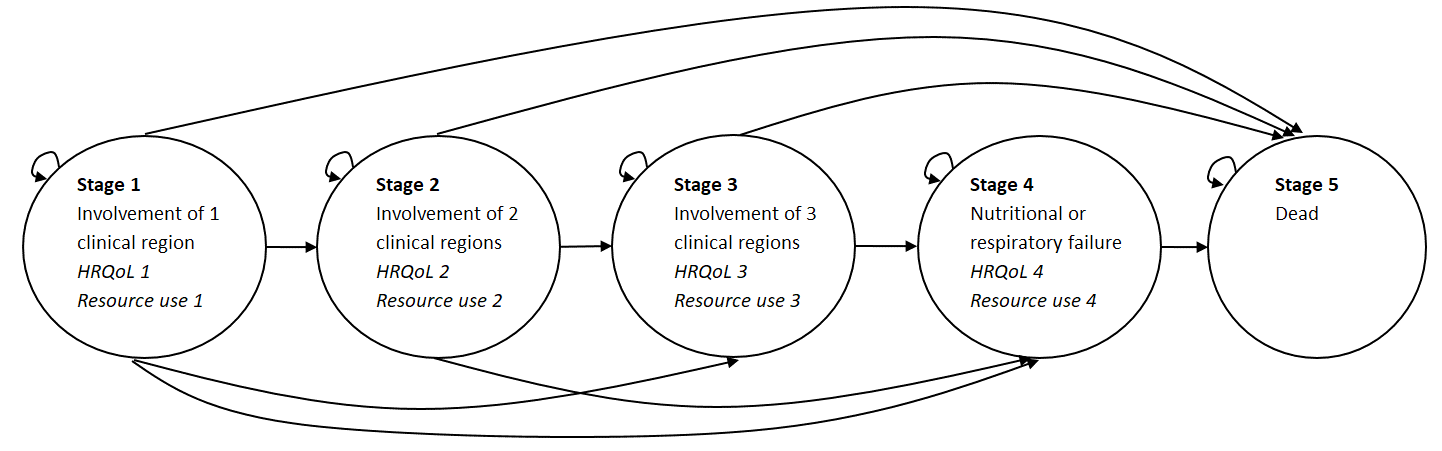


To estimate the treatment effects beyond the end of the trial, two scenarios would be modelled. First, it was assumed that any psychological benefit experienced would be sustained beyond the end of the trial and the increased QALY associated with psychological benefit will persist for as long the person stays alive. The second scenario assumed that the psychological benefit lasts for the duration of the intervention only. These two scenarios provided the most optimistic and pessimistic scenarios.

The following assumptions were made:

- All patients enter the model in Stage 2. This assumption was made by Tappenden et al [2023], based on clinical opinion and it will be tested based on baseline staging data and necessary modification will be made as appropriate. This assumption is realistic as in the trial the mean stage in which most participants entered was 2.3.
- During any model cycle, from any alive state, patients can only skip stages forward (patients do not get to a less severe state).
- Treatment response occurs at month 2 given the duration of the intervention.

### Methods for identifying and estimating parameters

The model required 4 main set of parameters: a. transition probabilities between health states b. treatment effects of the intervention group c. utility decrements d. costs. The transition probabilities from Tappenden et al [2023) were used. Costs from the trial were used. The utilities at the end of the trial period were extrapolated for the whole lifetime by using established methods for age adjustments.

### Model uncertainty

A probabilistic sensitivity analysis (PSA) was undertaken and the distributions for the model parameters was chosen based on within trial analysis. Assumptions will be made in assigning different distributions. A discount rate of 3.5% will be used based on NICE reference case. The PSA ran for 5,000 iterations to address uncertainty associated with the parameter estimates used to populate the model.

## **Results**

1. Pessimistic scenario where treatment effects only last for the study period

**Input parameters**

| **User setting** | **Selected option** |
| --- | --- |
| Model mode (1=deterministic; 2=PSA) | 1 |
| Discount rate – QALYs | 0.0% |
| Discount rate – costs | 0.0% |
| PPP | 1.01 |
| Age-adjust utility? (1=yes) | Yes |
| Initial distribution | All start in King's 2 |
| Proportion social care costed | 77.7% |
| Treatment effect - probability reduction | 0.00 |
| Utility gain on treatment | 0.02 |
| Intervention cost per cycle | £1,019 |
| Treat up to King's stage (inclusive) | 4 |
| Treatment effect duration (months) | 9 |
| Treatment cost duration (months) | 1 |
| Include indirect costs? | No |

**King’s staging – deterministic results**

| **Outcome** | **Comparator group** | **Intervention group** | **Incremental** |
| --- | --- | --- | --- |
| LYGs* | 2.23 | 2.23 | 0.00 |
| QALYs | 0.73 | 0.74 | 0.01 |
| Costs | £67,616 | £68,635 | £1,019 |
| ICER | - | - | **£96,589** |
| **Undiscounted* |  |  |  |

**LYG – life years gained; ICER: Incremental cost-effectiveness ratio; QALY: quality-adjusted life years gained**

**King’s staging – probabilistic results**

| **Outcome** | **Comparator group** | **Intervention group** | **Incremental** |
| --- | --- | --- | --- |
| LYGs* | 2.27 | 2.27 | .000 |
| QALYs | 0.75 | 0.74 | 0.01 |
| Costs | £68,047 | £69,066 | £1,019 |
| ICER | - | - | **£96,654** |
| **Undiscounted* |  |  |  |

**LYG – life years gained; ICER: Incremental cost-effectiveness ratio; QALY: quality-adjusted life years gained**

1. Optimistic scenario where treatment effects last for five years

**Input parameters**

| **User setting** | **Selected option** |
| --- | --- |
| Model mode (1=deterministic; 2=PSA) | 1 |
| Discount rate – QALYs | 0.0% |
| Discount rate – costs | 0.0% |
| PPP | 1.01 |
| Age-adjust utility? (1=yes) | Yes |
| Initial distribution | All start in King's 2 |
| Proportion social care costed | 77.7% |
| Treatment effect - probability reduction | 0.00 |
| Utility gain on treatment | 0.02 |
| Intervention cost per cycle | £1,019 |
| Treat up to King's stage (inclusive) | 4 |
| Treatment effect duration (months) | 60 |
| Treatment cost duration (months) | 1 |
| Include indirect costs? | No |

**King’s staging – deterministic results**

| **Outcome** | **Comparator group** | **Intervention group** | **Incremental** |
| --- | --- | --- | --- |
| LYGs* | 2.23 | 2.23 | 0.00 |
| QALYs | 0.73 | 0.76 | 0.03 |
| Costs | £67,616 | £68,635 | £1,019 |
| ICER | - | - | **£32,649** |
| **Undiscounted* |  |  |  |

**LYG – life years gained; ICER: Incremental cost-effectiveness ratio; QALY: quality-adjusted life years gained**

**King’s staging – probabilistic results**

| **Outcome** | **Comparator group** | **Intervention group** | **Incremental** |
| --- | --- | --- | --- |
| LYGs* | 2.27 | 2.27 | 0.00 |
| QALYs | 0.75 | 0.78 | 0.03 |
| Costs | £68,047 | £69,066 | £1,019 |
| ICER | - | - | **£32,314** |
| **Undiscounted* |  |  |  |

**LYG – life years gained; ICER: Incremental cost-effectiveness ratio; QALY: quality-adjusted life years gained**

# **Tables**

**Table A1a Unit costs for Primary and Community Services**

| **Primary and Community services** | Source | **Unit cost metric** | **Description** | **Unit cost £** |
| --- | --- | --- | --- | --- |
| GP | PSSRU 2022 | Minute | £41 per surgery consultation | 4.42 |
| Physiotherapist | NHS Costs 2021/22 | visit | Adult, One to One | 73 |
| Occupational therapist | NHS Costs 2021/22 | visit | Adult, One to One | 99 |
| Speech & language therapist | NHS Costs 2021/22 | visit | Adult, One to One | 128.16 |
| Dietician | NHS Costs 2021/22 | visit | Adult, One to One | 77.41 |
| Nutrition nurse | NHS Costs 2021/22 | visit | Other Specialist Nursing | 76 |
| Social worker | PSSRU 2022 | minute | £50 per hour | 0.83 |
| MNDA advisor ^a^ | PSSRU 2022 | minute | £50 per hour | 0.83 |
| MNDA volunteer visitor ^a^ | PSSRU 2022 | minute | £33 per hour | 0.55 |
| Psychologist / psychotherapist | PSSRU 2022 | visit | Adult, One to One | 66 |
| Community mental health team | NHS Costs 2021/22 | visit | Unit costs vary | 69 |
| Home help (household tasks) ^a^ | PSSRU 2022 | minute | £25 per hour | 0.417 |
| Home help (personal care) | PSSRU 2022 | minute | £25 per hour | 0.417 |
| Palliative care nurse | NHS Costs 2021/22 | visit | Adult, Face to face | 118 |
| MND nurse specialist | NHS Costs 2021/22 | visit | Specialist Nursing, Palliative/Respite Care | 118 |
| Respiratory nurse specialist | NHS Costs 2021/22 | visit | Specialist Nursing, Asthma and Respiratory Nursing | 110 |
| District nurse | NHS Costs 2021/22 | minute | face to face | 0.9 |
| Sitting service (charity provider) ^a^ | PSSRU 2022 | minute | £33 per hour | 0.55 |
| Counselling | PSSRU 2022 | visit | Average of Bands 5, 6 and 7 | 54 |

Notes: ^a^ These were included in the societal analysis only

**Table A1b Unit costs for Hospital, nursing home or hospice inpatient services**

|  | **Source** | **Unit cost metric** | **Description** | **Unit cost** |
| --- | --- | --- | --- | --- |
| Nursing or residential home | PSSRU 2022 | per day | establishment cost plus personal living expenses and external services per permanent resident per day | 181 |
| Hospice (including for respite care) | PSSRU 2022 | per day | establishment cost plus personal living expenses and external services per permanent resident per day | 181 |
| Intensive care unit | NHS Costs 2021/22 | FCE | Critical care | 1753 |
| Admission for gastrotomy tube insertion/management | NHS Costs 2021/22 | FCE | Endoscopic Insertion of Gastrostomy Tube, 19 years and over | 1959.34 |
| Admission for NIV/IV assessment/management | NHS Costs 2021/22 | FCE | Non-Invasive Ventilation Support Assessment, 19 years and over | 828.11 |

**Table A1c Unit costs for Outpatient and day care services**

|  | **Source** | **Unit cost metric** | **Description/Assumption** | **Unit cost £** |
| --- | --- | --- | --- | --- |
| Neurology outpatient ward | NHS Costs 2021/22 | activity |  | 214 |
| Day care centre (hospice) | PSSRU 2022 | minute | £17 per hour for 65+ | 0.27 |
| A&E visit (without admission) | NHS Costs 2021/22 | visit |  | 354 |

**Table A1d Unit costs for Equipment**

| Resource | Source | Unit cost metric | Description | Unit cost |
| --- | --- | --- | --- | --- |
| Ankle/foot orthotic | Commercial website | unit | Assume use for 1 year only | 132.26 |
| Walking aid - cane | Commercial website | unit | Assume use for two years | 3.15 |
| Walking aid - zimmer/rollator | Commercial website | unit | Assume use for two years | 11.84 |
| Wheelchair - manual | PSSRU 2022 | unit | Annual cost (capital and maintenance) | 99.00 |
| Wheelchair - electric | PSSRU 2022 | unit | Annual cost (capital and maintenance) | 345.75 |
| Adapted car with wheelchair access | Commercial website | unit | Assume use for 5 years and resale value of £5000 | 4068.89 |
| Mobile arm support | Commercial website | unit | Assume use for 5 years | 299.00 |
| Lightwriter | Commercial website | unit | Assume use for 5 years | 66.44 |
| Speech amplifier | Commercial website | unit | Assume use for 5 years | 18.27 |
| Stairlift | PSSRU 2020 | unit | Annual cost (capital and maintenance) | 276.75 |
| Specialist cutlery/cups/plates | Commercial website | unit | Assume lasts for 2 years | 11.84 |
| Riser recliner chair | Commercial website | unit | Assume use for 5 years | 66.44 |
| Specialist bed | Commercial website | unit | Assume use for 5 years | 197.67 |
| Mattress elevator | Commercial website | unit | Assume use for 5 years | 35.78 |
| Hoist - bedroom/mobile | Commercial website | unit | Assume use for 5 years | 149.50 |
| Wash and dry toilet | Commercial website | unit | Assume use for 5 years | 49.83 |
| Bath hoist | Commercial website | unit | Assume use for 5 years | 33.22 |
| Neck support | Commercial website | unit | Assume used only for 1 year | 5.99 |
| Environmental controls/switch | Commercial website | unit | Assume use for 5 years | 7.47 |
| Breathing equipment (NIV) | Published paper^a^ | unit | Assume use for 5 years | 488.31 |
| Note: Commercial and specialist websites included: completecare.co.uk, care.co.uk, amazon.co.uk, alliedmobility.com, livingmadeeasy.co.uk, abilia.com | | | | |
| ^a^ https://thorax.bmj.com/content/thoraxjnl/early/2021/11/25/thoraxjnl-2021-217463.full.pdf | | | | |

**Table A1e Unit costs for Home adaptations**

| **Resource** | **Source** | **Unit cost metric** | **Description** | **Unit cost** |
| --- | --- | --- | --- | --- |
| Extension built | PSSRU 2020 | Annual cost |  | 4554.44 |
| Downstairs toilet installed |  | Annual cost |  | 1334.42 |
| Downstairs shower installed |  | Annual cost |  | 1334.42 |
| Wheelchair ramps installed |  | Annual cost |  | 43.05 |
| Doors widened |  | Annual cost |  | 72.06 |
| Bathroom adapted |  | Annual cost |  | 629.78 |
| Through floor/lift/elevator |  | Annual cost | Weighted average of straight and more complex stairlift | 345.30 |
| Hand rails installed |  | Annual cost | Mean of internal and external | 4.66 |

Note: All costs have been calculated for 9 months and have been adjusted for inflation

**Table A1f Unit costs for Psychological Therapies**

| **Resource** | **Source** | **Unit cost metric** | **Description** | **Unit cost** |
| --- | --- | --- | --- | --- |
| CBT | PSSRU 2022 | minute | Average of Band 6 and 7 | 1.02 |
| Relaxation therapy |  | minute | Band 6 | 0.92 |
| MBCT or MBSR |  | minute | Average of Band 6 and 7 | 1.02 |
| Counselling |  | minute | Average of Bands 5, 6 and 7 | 0.90 |
| Psychodynamic therapy |  | minute | Band 7 | 1.10 |
| Not sure of therapy type |  | minute | Average of Band 6 and 7 | 0.98 |

**Table A2 EQ-5D-5L Anxiety and depression domain (% of participants)**

| Level | Baseline % | | 6 months | | 9 months | |
| --- | --- | --- | --- | --- | --- | --- |
|  | Intervention | Control | Intervention | Control | Intervention | Control |
| 1 | 46 | 48 | 56 | 45 | 49 | 43 |
| 2 | 29 | 33 | 31 | 41 | 36 | 39 |
| 3 | 23 | 12 | 12 | 10 | 12 | 12 |
| 4 | 2 | 2 | 1 | 3 | 3 | 4 |
| 5 | 0 | 1 | 0 | 1 |  | 1 |

**Table A3 Mean resource use by treatment arm**

|  | Intervention | Control |  |
| --- | --- | --- | --- |
|  | Mean [95% CI] | Mean [95% CI] | p value |
| Primary and community services | 31.38 [20.68 to 42.09] | 29.14 [11.00 to 47.27] | 0.19 |
| Hospital, nursing home or hospice services (visits) | 0.27 [0.13 to 0.41] ] | 0.26 [0.12 to 0.39] | 0.99 |
| Hospital, nursing home or hospice services (total nights ) | 1.50 [0.36 to 2.65] | 0.92 [0.32 to 1.51] | 0.85 |
| Outpatient and day care services (number of of attendances) | 1.64 [1.17 to 2.16] | 2.48 [1.45 to 4.23] | 0.26 |
| Equipment (NHS, LA, charities and self) | 3.84 [3.03 to 4.66] | 3.49 [2.81 to 4.16] | 0.91 |
| Home adaptations | 1.30 [0.82 to 1.77] | 0.86 [0.58 to 1.14] | 0.69 |
| Psychological therapies | 2.40 [0.76 to 4.04] | 0.79 [0.28 to 1.30] | 0.22 |

**Table A4 Resource use**

|  | **Intervention** | **Control** |
| --- | --- | --- |
| **Primary and community services** | **Number of visits** | |
| GP | 75 | 68 |
| Physiotherapist | 84 | 74 |
| Occupational therapist | 87 | 82 |
| Speech & language therapist | 65 | 61 |
| Dietician | 42 | 37 |
| Nutrition nurse | 12 | 8 |
| Social worker | 10 | 8 |
| MNDA advisor | 32 | 29 |
| MNDA volunteer visitor | 27 | 20 |
| Psychologist / psychotherapist | 34 | 18 |
| Community mental health team | 0 | 2 |
| Home help (household tasks) | 13 | 13 |
| Home help (personal care) | 11 | 14 |
| Palliative care nurse | 15 | 17 |
| MND nurse specialist | 57 | 58 |
| Respiratory nurse specialist | 42 | 38 |
| District nurse | 19 | 26 |
| Sitting service (charity provider) | 2 | 2 |
| Counselling | 6 | 13 |
| Alternative therapist (e.g. homeopathy) | 12 | 11 |
| Other | 31 | 50 |
| **Hospital, nursing home or hospice services** | **Number of visits** | |
| Nursing or residential home | 1 | 0 |
| Hospice (including for respite care) | 4 | 2 |
| Neurology inpatient ward | 2 | 2 |
| Admission for gastrostomy tube insertio | 9 | 8 |
| Admission for NIV / IV assessment / man | 2 | 8 |
| Other - please specify | 3 | 2 |
| **Outpatient and day care services** | **Number of attendances** | |
| Neurology outpatient ward | 42 | 47 |
| Day care centre (hospice) | 3 | 12 |
| A&E visit (without admission) | 11 | 8 |
| hospital outpatient visit – pleas | 41 | 42 |
| **Equipment** | **Have you acquired these?** | |
| Ankle / foot orthotic | 36 | 40 |
| Walking aid – cane | 22 | 17 |
| Walking aid - zimmer / rollator | 25 | 31 |
| Wheelchair – manual | 41 | 27 |
| Wheelchair – electric | 26 | 29 |
| Adapted car with wheelchair access | 17 | 7 |
| Mobile arm support | 7 | 4 |
| Lightwriter | 0 | 1 |
| Speech amplifier | 3 | 2 |
| Stairlift | 17 | 21 |
| Specialist cutlery / cups / plates | 21 | 15 |
| Riser recliner chair | 33 | 26 |
| Specialist bed | 16 | 13 |
| Mattress elevator | 7 | 5 |
| Hoist - bedroom / mobile | 12 | 9 |
| Wash and dry toilet | 15 | 6 |
| Bath hoist | 0 | 3 |
| Neck support | 11 | 11 |
| Specialist computer equipment | 10 | 7 |
| Environmental controls switch | 3 | 1 |
| Feeding pump (gastrostomy) | 6 | 6 |
| Breathing equipment (e.g. NIV) | 18 | 22 |
| Other please specify | 27 | 25 |
| **Home adaptations** | **Have you had a home adaptation?** | |
| Extension built | 1 | 0 |
| Downstairs toilet installed | 2 | 1 |
| Downstairs shower installed | 3 | 5 |
| Wheelchair ramps installed | 4 | 1 |
| Doors widened | 2 | 6 |
| Bathroom adapted | 3 | 0 |
| Through floor lift / elevator | 1 | 0 |
| Hand rails installed | 13 | 21 |
| Other | 6 | 2 |
| **Psychological therapies** | **Number of visits** | |
| CBT | 1 | 2 |
| Relaxation therapy | 5 | 1 |
| Mindfulness-Based CBT or Mindfulness-ba | 10 | 0 |
| Counselling | 6 | 11 |
| Psychodynamic therapy | 0 | 1 |
| Not sure of therapy type | 1 | 0 |
| Other - please specify | 2 | 3 |

**Table A5a Mean resource use costs - (with complete data n = 134)**

| **NHS and social care costs** | **Intervention** | | | **Control** | | | **Total** | | |
| --- | --- | --- | --- | --- | --- | --- | --- | --- | --- |
|  | N | Mean £ | SD £ | n | Mean £ | SD £ | n | Mean £ | SD £ |
| Primary and community services | 67 | 1754 | 1574 | 63 | 1666 | 2830 | 130 | 1711 | 2272 |
| Hospital, nursing home, hospice, inpatient | 10 | 496 | 1395 | 14 | 622 | 2134 | 24 | 558 | 1792 |
| Outpatient and day care services | 43 | 325 | 389 | 45 | 485 | 811 | 88 | 404 | 636 |
| Equipment costs | 52 | 365 | 432 | 45 | 427 | 620 | 97 | 395 | 532 |
| Home adaptation costs | 18 | 34 | 173 | 17 | 34 | 138 | 35 | 34 | 156 |
| Psychological therapies | 10 | 109 | 468 | 8 | 35 | 123 | 18 | 72 | 344 |
| **Total NHS resource use costs** | **68** | **3082** | **2826** | **65** | **3268** | **3971** | **133** | **3173** | **3426** |

**Table A5b Mean resource use costs – charities and out of pocket (with complete data n = 134)**

| **Charity and out of pocket expenditure** | **Intervention** | | | **Control** | | | **Total** | | |
| --- | --- | --- | --- | --- | --- | --- | --- | --- | --- |
|  | n | Mean £ | SD £ | n | Mean £ | SD £ | n | Mean £ | SD £ |
| Primary and community services | 67 | 2025 | 1818 | 65 | 1896 | 3168 | 132 | 1961 | 2564 |
| Equipment costs | 57 | 1380 | 2044 | 50 | 1014 | 1855 | 107 | 1200 | 1954 |
| Home adaptation costs | 32 | 885 | 2480 | 33 | 290 | 972 | 65 | 592 | 1910 |
| Psychological therapies | 13 | 133 | 482 | 11 | 53 | 152 | 24 | 94 | 360 |
| **Total costs by charities and out of pocket** | **68** | **4423** | **4476** | **66** | **3253** | **4038** | **134** | **3847** | **4290** |

**Table A6a Mean resource use costs – NHS and local authorities (with MI)**

| **NHS and social care costs** | **Intervention** | |  | **Control** | |  |
| --- | --- | --- | --- | --- | --- | --- |
|  | Mean £ | SD £ | CI | Mean £ | SD £ | CI |
| Primary and community services | 2076 | 1796 | 2041 to 2112 | 1721 | 2481 | 1670 to 1771 |
| Hospital, nursing home, hospice, inpatient | 823 | 2000 | 784 to 863 | 678 | 2080 | 636 to 720 |
| Outpatient and day care services | 341 | 398 | 333 to 349 | 481 | 744 | 466 o 496 |
| Equipment costs | 447 | 491 | 437 to 457 | 468 | 618 | 456 to 481 |
| Home adaptation costs | 38 | 176 | 35 to 42 | 30 | 126 | 27 to 32 |
| Psychological therapies | 112 | 444 | 103 to 120 | 35 | 119 | 33 to 37 |
| **Total NHS resource use costs** | 3838 | 3729 | 3764 to 3912 | 3412 | 3629 | 3338 to 3485 |

**Table A6b Mean resource use costs – NHS and local authorities (with MI)**

| **Charity and out of pocket expenditure** | **Intervention** | |  | **Control** | |  |
| --- | --- | --- | --- | --- | --- | --- |
|  | Mean £ | SD £ | CI | Mean £ | SD £ | CI |
| Primary and community services | 2025 | 1805 | 1982 to 2068 | 1896 | 3144 | 1820 to 971 |
| Equipment costs | 1380 | 2029 | 1332 to 1427 | 1014 | 1841 | 970 to 1058 |
| Home adaptation costs | 885 | 2462 | 827 to 943 | 290 | 965 | 267 to 313 |
| Psychological therapies | 133 | 478 | 122 to 144 | 53 | 151 | 49 to 57 |
| **Total costs by charities and out of pocket** | **4423** | **4443** | **4318 to 4527** | **3253** | **4007** | **3157 to 3350** |

**Table A7 Intervention costs**

|  | **Mean £** | **SE** | **95% CI** |
| --- | --- | --- | --- |
| Delivery of sessions | 503 | 22 | 460 to 546 |
| Supervision costs^a^ | 180 |  |  |
| Training costs | 29 | 0.3 | 29 to 30 |
| Total intervention costs | 712 | 22 | 669 to 756 |

^a^ The costs of supervision have been allocated equally to all participants in the intervention group

Subgroup analyses

Analysis will be conducted on the final dataset to investigate how cost-effectiveness varies between different patient subgroups. Heterogeneity will be explored if there is a statistically significant difference in the primary outcome, the McGill Quality of Life-Revised (MQOL-R). A p value of 0.1 instead of the usual p = 0.05 will be used in line with guidance from the European Medicines Agency [EMEA 1998] Furthermore, heterogeneity will be explored between subgroups and for subgroups of 50 participants or more. Subgroups that will be considered include: severity of condition at baseline using ALSFRS-R, severity of baseline depression and anxiety using Modified Hospital Anxiety and Depression Scale – depression component (M-HADS-D) and Modified Hospital Anxiety and Depression Scale – anxiety component (M-HADS-A), rate of deterioration and participants recruited during the COVID-19 pandemic versus those recruited pre and post the restrictions. We will use 3 subgroups with cut points for before and after the beginning of national lockdown on 23 March 2020 and before and after the final easing of lockdown restrictions on 27 January 2022. Separate subgroups between these dates will not be used because although the rules changed frequently during this period people did not begin to return to usual behaviours until the final easing on 27 January 2022.

However due to group sizes, subgroup analyses were carried out only by severity of condition and rate of deterioration or progression of disease.

**Table A8 Costs and QALYs (ITT with imputation)**

| Costs per participant | Intervention  *Mean* (SE)  (n = 97) | Control  *Mean (SE)*  (n = 94) | Mean difference  95% CI | p value |
| --- | --- | --- | --- | --- |
| **Primary analysis with imputed costs and QALYs** | | | | |
| Intervention costs | 712 (22) |  | 712 [668 to 756] | <0.001 |
| Resource use costs | 3843 (406) | 3413 (489) | -185 (-1361 to 990) | 0.755 |
| Total costs | 4555 (403) | 3413 (401) | 1142 [16 to 2269] | 0.047 |
| Utilities and QALYs |  |  |  |  |
| EQ-5D scores at baseline | 0.509 (0.029) | 0.533 (0.028) | -0.024 [-0.103 to 0.056] | 0.556 |
| EQ-5D scores at 6 months | 0.418 (0.036) | 0.427(0.034) | -0.008 [-0.106 to 0.089] | 0.864 |
| EQ-5D scores at 9 months | 0.379 (0.035) | 0.378 (0.036) | 0.000 [-0.098 to 0.099] | 0.996 |
| Total QALY at 9 months | 0.33 (0.02) | 0.34 (0.02) | -0.019 [-0.07 to 0.05] | 0.719 |

**Table A9 Primary analysis ITT with MI**

|  | **Mean difference** | **SE** | **95% Confidence Interval** | **P value** |
| --- | --- | --- | --- | --- |
| Total Cost (£) | 1019 | 537 | -34 to 2074 | 0.058 |
| QALYs | 0.012 | 0.015 | -0.019 to 0.042 | 0.456 |
| ICER | £88,507 | | |  |
| Probability that ACT is cost-effective | 8% | | |  |

**Secondary analysis with MQOL as outcome**

**Table A10 MQOL scores (ITT with imputation)^a^**

| Costs per participant | Intervention  *Mean* (SE)  (n = 97) | Control  *Mean (SE)*  (n = 94) | Mean difference  95% CI | p value |
| --- | --- | --- | --- | --- |
| MQOL-R scores at baseline | 6.68 (1.46) | 6.84 (1.39) | -0.16 [-0.57 to 0.25] | <0.001 |
| MQOL-R scores at 6 months | 6.84 (1.65) | 6.27 (1.60) | 0.57 [0.08 to 1.06] | <0.001 |
| MQOL-R at 9 months | 6.76 (1.60) | 6.18 (1.69) | 0.588 [0.07 to 1.111] | <0.001 |
| Total MQOL_R score 9 months | 5.08 (1.02) | 4.83 (1.05) | 0.25 [-0.06 to 0.56] | <0.001 |

**^a^ Please note that the MI conducted for the statistical analysis and the health economics has been conducted differently. Hence the figures may be different and this is not a problem as both sets of analyses are in line with the respective analysis plan.**

**Table A11 Secondary analysis – cost-effectiveness results**

|  | **Mean difference** | **SE** | **95% Confidence Interval** | **P value** |
| --- | --- | --- | --- | --- |
| Total Cost (£) | 1050 | 537 | -34 to 2074 | <0.001 |
| MQOL score over 9 month (area under the curve) | 0.34 | 0.09 | 0.15 to 0.52 | <0.001 |
| ICER | £3120 | | |  |
| Probability that ACT is cost-effective if NHS is willing to pay £5,000 for a point improvement in MQOL-R score | | | | 85 % |
| Probability that ACT is cost-effective if NHS is willing to pay £10,000 for a point improvement in MQOL-R score | | | | 100% |

**Table A12 Costs and QALYs (complete case)**

| Costs per participant | Intervention  *Mean* (SE)  (n = 68) | Control  *Mean (SE)*  (n = 65) | Mean difference  95% CI | p value |
| --- | --- | --- | --- | --- |
| **Primary analysis with imputed costs and QALYs** | | | | |
| Intervention costs | 790 (105) |  | 790 [764 to 816] | <0.001 |
| NHS resource use costs | 3872 (2802) | 3281 (4000) | 592 [-589 to 1772] | 0.323 |
| Utilities and QALYs |  |  |  |  |
| EQ-5D scores at baseline | 0.567 (0.225) | 0.592(0.228) | -0.025 [-0.102 to 0.053] | 0.529 |
| EQ-5D scores at 6 months | 0.491 (0.301) | 0.505 (0.269) | -0.014 [-0.112 to 0.084] | 0.778 |
| EQ-5D scores at 9 months | 0.445 (0.301) | 0.438 (0.313) | 0.008 [-0.098 to 0.113] | 0.885 |
| Total QALY at 9 months | 0.38 (0.19) | 0.39 (0.18) | -0.01 [-0.07 to 0.05] | 0.750 |

**Table A13 Complete case analysis - cost-effectiveness results**

|  | **Mean difference** | **SE** | **95% Confidence Interval** | **P value** |
| --- | --- | --- | --- | --- |
| Total Cost (£) | 522 | 570 | -596 to 1640 | 0.360 |
| QALYs | 0.008 | 0.015 | -0.022 to 0.036 | 0.624 |
| ICER | £72,233 | | |  |
| Probability that ACT is cost-effective | 26% | | |  |

**Figure A1 Confidence ellipse: Complete case analysis**

**Figure A2 Cost-effectiveness acceptability curve: complete case analysis**

**Table A14 Costs and QALYs (per protocol)**

| Costs per participant | Intervention  *Mean* (SE)  (n = 68) | Control  *Mean (SE)*  (n = 65) | Mean difference  95% CI | p value |
| --- | --- | --- | --- | --- |
| **Primary analysis with imputed costs and QALYs** | | | | |
| Intervention costs | 802 (11) |  | 802 [782 to 821] | <0.001 |
| NHS resource use costs | 3836 (349) | 3268 (489) | 568 [-630 to 1766] | 0.350 |
| Utilities and QALYs |  |  |  |  |
| EQ-5D scores at baseline | 0.541 (0.030) | 0.533 (0.028) | 0.008 [-0.073 to 0.089] | 0.845 |
| EQ-5D scores at 6 months | 0.448 (0.038) | 0.424 (0.034) | 0.025 [-0.076 to 0.126] | 0.628 |
| EQ-5D scores at 9 months | 0.422 (0.035) | 0.377 (0.036) | 0.045 [-0.056 to 0.145] | 0.385 |
| Total QALY at 9 months | 0.356 (0.025) | 0.342 (0.022) | 0.014 [-0.051 to 0.080] | 0.664 |

**Table A15 Per protocol analysis**

|  | **Mean difference** | **SE** | **95% Confidence Interval** | **P value** |
| --- | --- | --- | --- | --- |
| Total Cost (£) | 539 | 579 | -595 to 1664 | 0.352 |
| QALYs | 0.011 | 0.014 | -0.017 to 0.039 | 0.426 |
| ICER | £47,582 | | |  |
| Probability that ACT is cost-effective | 30% | | |  |

**Figure A3 Confidence ellipse: Per protocol analysis**

**Figure A4 Cost-effectiveness acceptability curve: per protocol analysis**

**Table A16 Costs and QALYs (ITT societal perspective with multiple imputation)**

| Costs per participant | Intervention  *Mean* (SE)  (n = 97) | Control  *Mean (SE)*  *(n = 94)* | Mean difference  95% CI | p value |
| --- | --- | --- | --- | --- |
| **Primary analysis with imputed costs and QALYs** | | | | |
| Intervention costs | 712 (22) |  | 712 [668 to 756] | <0.001 |
| Societal resource use costs | 9785 (794) | 6531 (697) | 3254 [1156 to 5353] | 0.003 |
| Utilities and QALYs |  |  |  |  |
| EQ-5D scores at baseline | 0.509 (0.029) | 0.533 (0.028) | -0.024 [-0.103 to 0.056] | 0.558 |
| EQ-5D scores at 6 months | 0.419 (0.036) | 0.426 (0.034) | -0.007 [-0.011 to 0.091] | 0.893 |
| EQ-5D scores at 9 months | 0.381 (0.035) | 0.381 (0.036) | -0.000 [-0.099 to 0.098] | 0.995 |
| Total QALY at 9 months | 0.332 (0.023) | 0.343 (0.022) | -0.011 [-0.074 to 0.052] | 0.734 |

**Table A17 ITT societal perspective with multiple imputation**

|  | **Mean difference** | **SE** | **95% Confidence Interval** | **P value** |
| --- | --- | --- | --- | --- |
| Total Cost (£) | 2927 | 973 | 1020 to 4834 | 0.003 |
| QALYs | 0.012 | 0.015 | -0.018 to 0.043 | 0.78 |
| ICER | £241,093 | | |  |
| Probability that ACT is cost-effective | <1% | | |  |

**Figure A5 Confidence ellipse: Partial societal analysis**

**Figure A6 Cost-effectiveness acceptability curve: Partial societal analysis**

**Figure A7 Confidence ellipse: Subgroup analysis – minimal to mild severity**

**Figure A8 Cost-effectiveness acceptability curve: Subgroup analysis – minimal to mild severity**

**Figure A9 Confidence ellipse: Subgroup analysis – mild to moderate severity**

**Figure A10 Cost-effectiveness acceptability curve: Subgroup analysis – mild to moderate severity**

**Table A18 Subgroups**

| Subgroup description | Subgroup categories | No of plwMND in subgroup |
| --- | --- | --- |
| 1. Severity of condition at baseline using ALS-FRS-R score | 40-48 (minimal to mild)  39-30 (mild to moderate)  20-29 (moderate to severe)  < 20 (advanced disease) | 58  99  31  3 |
| 1. Severity of baseline depression symptoms using M-HADS-D score | <8 (not case level depression)  >= 8 (case level depression) | 173  18 |
| 1. Severity of baseline anxiety symptoms using M-HADS-A score | <9 (not case level anxiety)  >= 9 (case level anxiety) | 150  41 |
| 1. Rate of deterioration or progression of disease | Lowest deterioration  Medium deterioration  Highest deterioration | 64  64  63 |
| 1. Timing of randomisation related to the COVID pandemic | Before 23^rd^ March 2020  23^rd^ March 2020 to 27^th^ Jan 2022  After 27^th^ Jan 2022 | 25  120  46 |

**Table A19 Subgroup analysis based on ALSFRS-R severity**

| 1. **Based on ALS-FRS-R severity Minimal to mild (n = 58)** | | | | |
| --- | --- | --- | --- | --- |
|  | **Mean difference** | **SE** | **95% Confidence Interval** | **P value** |
| Total Cost (£) | 23 | 794 | -1532 to 1579 | 0.977 |
| QALYs | 0.009 | 0.018 | -0.027 to 0.045 | 0.620 |
| ICER | £2570 | | |  |
| Probability that ACT is cost-effective | 58% | | |  |
| 1. **Based on ALSFRS-R severity Mild to moderate (n = 99)** | | | | |
|  | **Mean difference** | **SE** | **95% Confidence Interval** | **P value** |
| Total Cost (£) | 590 | 821 | -1021 to 2200 | 0.473 |
| QALYs | 0.013 | 0.023 | -0.032 to 0.058 | 0.575 |
| ICER | £45,972 | | |  |
| Probability that ACT is cost-effective | 35% | | |  |

**Table A20 Subgroup analysis based on rates of deterioration**

| 1. **Based on deterioration rate Lowest deterioration rate (n = 64)** | | | | |
| --- | --- | --- | --- | --- |
|  | **Mean difference** | **SE** | **95% Confidence Interval** | **P value** |
| Total Cost (£) | 626 | 635 | -619 to 1872 | 0.324 |
| QALYs | -0.008 | 0.020 | -0.047 to 0.032 | 0.696 |
| ICER | £79,528 | | |  |
| Probability that ACT is cost-effective | 12% | | |  |
| 1. **Based on deterioration rate Medium deterioration rate (n = 64)** | | | | |
|  | **Mean difference** | **SE** | **95% Confidence Interval** | **P value** |
| Total Cost (£) | -450 | 964 | -2339 to 1438 | 0.640 |
| QALYs | 0.033 | 0.023 | -0.013 to 0.078 | 0.164 |
| ICER | £13,817 | | |  |
| Probability that ACT is cost-effective | 86% | | |  |
| 1. **Based on deterioration rate Highest deterioration rate (n = 63)** | | | | |
|  | **Mean difference** | **SE** | **95% Confidence Interval** | **P value** |
| Total Cost (£) | 3291 | 1059 | 1213 to 5369 | 0.002 |
| QALYs | -0.011 | 0.034 | -0.078 to 0.056 | 0.744 |
| ICER | £ 293,618 | | |  |
| Probability that ACT is cost-effective | <1% | | |  |

**Figure A11 Confidence ellipse: Subgroup analysis – lowest deterioration rate**

**Figure A12 Cost-effectiveness acceptability curve: Subgroup analysis – lowest deterioration rate**

**Figure A13 Confidence ellipse: Subgroup analysis – medium deterioration rate**

**Figure A14 Cost-effectiveness acceptability curve: Subgroup analysis – medium deterioration rate**

**Figure A15 Confidence ellipse: Subgroup analysis – highest deterioration rate**

**Figure A16 Cost-effectiveness acceptability curve: Subgroup analysis – highest deterioration rate**
